# Unravelling the Bacterial Vaginosis-Associated Biofilm: A Multiplex *Gardnerella vaginalis* and *Atopobium vaginae* Fluorescence *In Situ* Hybridization Assay Using Peptide Nucleic Acid Probes

**DOI:** 10.1371/journal.pone.0136658

**Published:** 2015-08-25

**Authors:** Liselotte Hardy, Vicky Jespers, Nassira Dahchour, Lambert Mwambarangwe, Viateur Musengamana, Mario Vaneechoutte, Tania Crucitti

**Affiliations:** 1 Unit of Epidemiology and Control of HIV/STD, Department of Public Health, Institute of Tropical Medicine, Antwerp, Belgium; 2 Laboratory Bacteriology Research, Faculty of Medicine & Health Sciences, University of Ghent, Ghent, Belgium; 3 STI Reference Laboratory, Department of Clinical Sciences, Institute of Tropical Medicine, Antwerp, Belgium; 4 Plantijn Hogeschool, Antwerp, Belgium; 5 Rinda Ubuzima, Kigali, Rwanda; University Hospital of the Albert-Ludwigs-University Freiburg, GERMANY

## Abstract

Bacterial vaginosis (BV), a condition defined by increased vaginal discharge without significant inflammation, is characterized by a change in the bacterial composition of the vagina. *Lactobacillus* spp., associated with a healthy vaginal microbiome, are outnumbered by BV-associated organisms. These bacteria could form a polymicrobial biofilm which allows them to persist in spite of antibiotic treatment. In this study, we examined the presence of *Gardnerella vaginalis* and *Atopobium vaginae* in vaginal biofilms using Peptide Nucleic Acid (PNA) probes targeting these bacteria. For this purpose, we developed three new PNA probes for *A*. *vaginae*. The most specific *A*. *vaginae* probe, AtoITM1, was selected and then used in an assay with two existing probes, Gard162 and BacUni-1, to evaluate multiplex FISH on clinical samples. Using quantitative polymerase chain reaction (qPCR) as the gold standard, we demonstrated a sensitivity of 66.7% (95% confidence interval: 54.5% - 77.1%) and a specificity of 89.4% (95% confidence interval: 76.1% - 96%) of the new AtoITM1 probe. FISH enabled us to show the presence of a polymicrobial biofilm in bacterial vaginosis, in which *Atopobium vaginae* is part of a *Gardnerella vaginalis*-dominated biofilm. We showed that the presence of this biofilm is associated with high bacterial loads of *A*. *vaginae* and *G*. *vaginalis*.

## Introduction

Bacterial vaginosis (BV), a condition characterized by increased vaginal discharge without significant inflammation, is highly prevalent in women of reproductive age. It increases the risk for acquisition and transmission of sexually transmitted infections, including HIV, and is associated with preterm birth in pregnant women [1–2]. BV is a dysbiotic condition of unknown etiology and is characterized by a change in the microbial composition of the vagina. *Lactobacillus* spp., associated with a healthy vaginal microbiome, are outnumbered by an array of BV-associated organisms including *Gardnerella vaginalis* [1–3]. However, several studies suggest that the mere presence of *G*. *vaginalis* is not sufficient for the diagnosis of BV. Indeed, *G*. *vaginalis* is also present in 50% to 70% of women without BV according to Nugent score [4–6]. *G*. *vaginalis* expresses various virulence factors such as vaginolysin [7] and sialidase [8]. It can also produce a biofilm [9], thereby increasing its tolerance to lactic acid and hydrogen peroxide produced by lactobacilli [10,11] and to antimicrobial treatment [12,13]. Furthermore, it has been suggested that its adherence and biofilm-forming capacities allow *G*. *vaginalis* to initiate the colonization and scaffolding of the vaginal epithelium to which other species can attach subsequently [14,15].

As was first shown a decade ago, *Atopobium vaginae* is one of the many other species that are characteristic of BV [16–20]. In one study, *A*. *vaginae* was detected in 80% of samples testing positive for *G*. *vaginalis* and made up 40% of the total biofilm mass dominated by *G*. *vaginalis* [9]. This association was confirmed in a study by Bradshaw et al. [21]: 93% of samples containing *A*. *vaginae* also contained *G*. *vaginalis*, whereas only 10% tested positive for *G*. *vaginalis* when *A*. *vaginae* was absent [22]. In contrast to *G*. *vaginalis*, *A*. *vaginae* is rarely part of the healthy vaginal microbiome and is considered a more specific marker of BV than *G*. *vaginalis* [17,21,23].

It is postulated that a biofilm provides bacteria with a competitive advantage over planktonic bacteria and that polymicrobial biofilms may offer additional advantages over single-species biofilms. Mechanisms that have been described in previous studies include metabolic cooperation, increased resistance to antibiotics or host immune responses [24] and an enlarged gene pool with more efficient sharing of genetic material compared to mono-species biofilms [25]. Polymicrobial coexistence is the dominant form in environmental biofilms, but is also prominent in the human body [24]. A well-known example is dental plaque: anaerobic bacteria, which are sensitive to oxygen, can survive and persist under the aerobic conditions in the oral cavity due to the consummation of oxygen by aerobic bacteria in the dental biofilm [26].

On the basis of these previous findings, we hypothesize that a polymicrobial biofilm consisting of *A*. *vaginae* and *G*. *vaginalis* and other bacteria not discussed in this study may serve as a marker of BV. Thus, better visualization of the structure of vaginal biofilms and identification of the bacterial components of the biofilm may contribute to better understanding of BV. To study the role of *A*. *vaginae* and *G*. *vaginalis* in BV, we designed and evaluated the performance of fluorescence *in situ* hybridization (FISH) with peptide nucleic acid (PNA) probes for *A*. *vaginae* and *G*. *vaginalis*. Three new PNA probes were designed for *A*. *vaginae*. The most specific *A*. *vaginae* probe was selected, and subsequently used together with a PNA probe that had been described for the detection of *G*. *vaginalis* and a positive control probe that detects a broad range of bacteria in order to evaluate the multiplex FISH on clinical samples.

## Materials and Methods

### Design of PNA probes

PNA probes targeting the bacterial 16S rRNA were synthesized by Panagene (Daejeon, South Korea). A fluorescent label was attached using a double 8-amino-3,6-dioxaoctanoic acid (AEEA) linker at the N terminus. We evaluated the performance of three new PNA probes for *A*. *vaginae*: Ato291, previously described as a DNA FISH probe [9,27] and AtoITM1 and AtoITM2, which we developed using the Applied Biosystems PNA designer software (http://www6.appliedbiosystems.com/support/pnadesigner.cfm), based on sequences of species-specific PCR primers from Burton [28] for AtoITM1 and from Fredricks [29] for AtoITM2. The probes met the following criteria: purine content was limited to less than 60%; a maximum of four purines in a purine-stretch and a maximum of three guanines in a guanine-stretch were allowed; and self-complementarity was absent, considering that PNA/PNA interactions are stronger than PNA/DNA interactions. For the detection of *G*. *vaginalis*, a previously described probe, Gard162 [30,31], was used. The broad-range bacterial probe, BacUni-1, previously designed [32] as a modified version of the broad-range eubacterial DNA probe [33], was used as a positive control. The probe specifications are listed in Table 1.

**Table 1:** Probe specifications.

Name	Target	Probe sequence (5’-3’)	Tm (°C)	%GC	Reference
AtoITM1	*A*. *vaginae*	Alexa488-OO-CTC-CTG-ACC-TAA-CAG-ACC	66	55.6	Newly designed, based on Burton et al. [28]
AtoITM2	*A*. *vaginae*	Alexa488-OO-GCG-GTY-TGT-TAG-GTC-AGG	72	58.3	Newly designed, based on Fredricks et al. [29]
Ato291	*A*. *vaginae*	Alexa488-OO-GGT-CGG-TCT-CTC-AAC-CC	68	60.0	Newly designed, based on Harmsen et al. [27]
Gard162	*G*. *vaginalis*	Alexa647-OO-CAG-CAT-TAC-CAC-CCG	61	60.0	Machado et al. [30]
BacUni-1	Eubacteria	Alexa555-CTG-CCT-CCC-GTA-GGA	64	66.7	Perry-O'Keefe et al. [32]

### Bacterial culture techniques for evaluation performance PNA probes

The performance of the PNA probes was evaluated using clinical isolates, obtained from the collections of the Institute of Tropical Medicine (ITM) and Ghent University. The five most frequently detected *Lactobacillus* species [3,34], representing the non-BV microbiome, were chosen as a negative control to assure that the probes would not cross-hybridize with the normal microbiome (Table 2). Furthermore we selected the most frequent BV-associated bacteria (8 *A*. *vaginae* strains, 5 *G*. *vaginalis* strains) as a negative control for *G*. *vaginalis* and *A*. *vaginae* respectively in addition to *2 Prevotella melaninogenica* strains (Table 2). This small test panel is a limitation of the study and more expansive testing will be required to assure that there is no cross-reactivity with other bacteria. The strains from frozen stocks in skimmed milk (-80°C) were cultured on Columbia agar base (Becton Dickinson Biosciences, Erembodegem, Belgium) + 5% horse blood and grown under anaerobic conditions (10% hydrogen, 10% carbon dioxide and 80% nitrogen), using an anaerobic incubator (Whitley DG250) at 37°C for 48 h and bacteria were streaked onto fresh plates every 48–72 hours. To determine the limit of detection, FISH was performed using the AtoITM1, Gard162 and BacUni-1 probe on serial tenfold dilutions using the fixative used throughout the study: Carnoy solution (6:3:1, ethanol:chloroform:glacial acetic acid [35,36]). Carnoy solution was chosen as a fixative solution because of its proven effectiveness in the stabilization of and minimal shrinkage in tissue structure [36,37]. The concentration of the dilutions was estimated based on the turbidity of the sample compared to McFarland Standards (Bio-Merieux SA, Marcy l’Etoile, France) and ranged from 1.2x10^9^ to 1.2x10^3^ cells per ml. The suspensions were vortexed briefly and 5 μl was spotted into the hybridization chamber; a 5 mm² area marked with a PAP Pen (Sigma Aldrich, St. Louis, USA) that creates a hydrophobic border on a Superfrost Plus slide (Menzel-Gläser, Braunschweig, Germany). The spotted suspensions were dried before performing FISH. Experiments were performed in duplicate.

**Table 2:** Specificity testing in duplicate of PNA probes using cultured bacteria. The signal was considered positive if it had a positive counterpart in the DAPI stain and displayed a positive signal simultaneously with the broad-range probe. The signal was considered negative if no signal was seen with the species-specific probe.

Species	Strain	AtoITM1	AtoITM2	Ato291	Gard162	BacUni-1
*Atopobium vaginae*	CCUG 38953^T^	+^1^	+	+	-^2^	+
*Atopobium vaginae*	UG080499	+	+	+	-	+
*Atopobium vaginae*	UG071164	+	+	+	-	+
*Atopobium vaginae*	UG020349	+	+	+	-	+
*Atopobium vaginae*	UG160373	+	+	+	-	+
*Atopobium vaginae*	UG550940	+	+	+	-	+
*Atopobium vaginae*	UG030313	+	+	+	-	+
*Atopobium vaginae*	UG030312	+	+	+	-	+
*Gardnerella vaginalis*	UG860108	-	-	-	+	+
*Gardnerella vaginalis*	UG030406	-	-	-	+	+
*Gardnerella vaginalis*	UG860107	-	+	+	+	+
*Gardnerella vaginalis*	LMG 7832^T^	-	+	+	+	+
*Gardnerella vaginalis*	UG030407	-	+	+	+	+
*Lactobacillus iners*	LMG 18914^T^	-	-	+	-	+
*Lactobacillus vaginalis*	LMG 12891^T^	-	-	+	-	+
*Lactobacillus jensenii*	LMG 6414^T^	-	-	+	-	+
*Lactobacillus crispatus*	LMG 9479^T^	-	-	+	-	+
*Lactobacillus gasseri*	LMG 9203^T^	-	-	+	-	+
*Prevotella melaninogenica*	UG160361	-	-	+	-	+
*Prevotella melaninogenica*	UG040818	-	-	-	-	+

^1^(+) Presence of hybridization

^2^(-) Absence of hybridization.

### Clinical samples

#### Ethics statement

Vaginal samples were collected from 119 women participating in a clinical trial in Rwanda studying the vaginal microbiome and acceptability of a contraceptive ring (S1 Protocol) (the ‘Ring Plus’ study, ClinicalTrials.gov identifier NCT01796613) (data analysis on-going) [38]. Participants were between 18 and 35 years old and provided written informed consent for participation in the study. The Ring Plus study and consent procedure were approved by the Rwanda National Ethics Committee, Rwanda; the Institutional Review Board of the ITM Belgium; and the ethics committee of the University Teaching Hospital in Antwerp, Belgium.

#### Vaginal sample collection and preparation

Vaginal sampling was carried out by the study clinician as part of the study procedures. Two Copan flocked swabs (Copan, Brescia, Italy) and one cotton swab were brushed against the lateral walls of the vagina. The cotton swab was immediately rolled on a Superfrost Plus slide (Menzel-Gläser) which was heat-fixed by passing twice through a flame. The Superfrost Plus (Menzel-Gläser) slides were stored for maximum six months and shipped to ITM at room temperature and fixed for a minimum of 12 hours at ITM, submerged in Carnoy solution [35,36]. The Copan flocked swabs were eluated by vortexing each swab for at least 15 seconds in 1.2 ml of diluted phosphate buffered saline (PBS) (pH 7.4 - 1:9, PBS:saline). The two eluates were combined and divided into three aliquots, which were stored at -80°C. The swab eluates were shipped frozen (-191°C) in a dry shipper to the ITM to determine the total bacterial load of *A*. *vaginae* and *G*. *vaginalis* by means of quantitative real-time polymerase chain reaction (qPCR).

#### Urine sample collection and preparation

According to an earlier described procedure [39], first-void urine was collected by the participants and 2 ml was transferred immediately to a 15 ml tube containing 2 ml of Carnoy solution. The sample was fixed overnight and after centrifugation (10 minutes at 3200 g), the supernatant was decanted and the pellet was treated for a second time with 0.75 μl of Carnoy solution. The samples were stored between 2–8°C and shipped at room temperature to the ITM. Before applying FISH, the urine samples were vortexed briefly and 5 μl was spotted into the hybridization chamber on a Superfrost Plus slide (Menzel-Gläser).

#### Quantitative PCR for quantification of bacteria in vaginal samples

DNA was extracted from 250 μl of the vaginal swab eluate using the Abbott m2000sp automated extraction platform (Abbott, Maidenhead, UK), according to the manufacturer’s instructions. The volume of 200 μl DNA extract was stored at –80°C until testing. qPCR was performed for each bacteria species separately, to avoid competition between the primers. The 25 μl PCR mixture contained 12.5 μl Rotor-Gene SYBR Green RT-PCR Master mix (Qiagen, Venlo, the Netherlands), 5 μl DNA extract, 0.5 μM of *A*. *vaginae* or 1 μM of *G*. *vaginalis* forward and reverse primers (Integrated DNA Technologies, Leuven, Belgium) and RNase-free water provided with the Rotor-Gene SYBR Green PCR kit. The primers for *A*. *vaginae* and *G*. *vaginalis* and the amplification reactions (Rotor Gene Q MDx 5 plex, Qiagen) have been described before [34].

Quantification was done using standard curves, constructed using DNA extracts from *A*. *vaginae* (CCUG 38953^T^) and *G*. *vaginalis* (LMG 7832^T^), grown at 35°C ± 2°C on Columbia agar base (Becton Dickinson) + 5% horse blood, under anaerobic conditions. DNA concentrations were determined using NanoDrop (Thermo Fisher Scientific, Erembodegem, Belgium) and the number of genomes was calculated using the described genome sizes and G+C content of the strains. A total of six tenfold dilutions of the DNA stocks were prepared in high performance liquid chromatography (HPLC) grade water. Both the standard curve and samples were run in duplicate. The bacterial load was expressed as genome equivalents (geq)/ml.

### PNA FISH procedure

Multiplex hybridization was performed on a Superfrost Plus slide (Menzel-Gläser) in a 5 mm² quadrant hybridization area marked with a PAP pen (Sigma Aldrich, St. Louis, USA), a liquid-repellent slide marker. The slide was covered with a cover slip after addition of a hybridization buffer that contained 200 nM of each probe: species-specific probes for *A*. *vaginae* (AtoITM1 or AtoITM2 or Ato291) and *G*. *vaginalis* (Gard162), and the broad-range BacUni-1 probe. The hybridization solution consisted of 10% (wt/vol) dextran sulphate (Sigma Aldrich), 10 mM NaCl (Merck KGaA, Darmstadt, Germany), 2% (vol/vol) formamide (Merck KGaA), 0.1% (wt/vol) sodium pyrophosphate (Sigma Aldrich), 0.2% (wt/vol) polyvinylpyrrolidone (Sigma Aldrich), 0.2% (wt/vol) Ficoll (Sigma Aldrich), 5 mM disodium EDTA (Merck KGaA), 0.1% (vol/vol) Triton X-100 (Acros Organics, Geel, Belgium) and 50 mM Tris-HCl at pH 7.5 (Sigma Aldrich).

The slides were incubated in a hybridization oven (Shake ‘N Bake, Boekel Scientific, Feasterville, Pennsylvania) in humid conditions, which were achieved by adding a small tray of water, at 60°C for 60 minutes. After the slides were rinsed with double-distilled (dd) H_2_0, they were immersed in a washing solution containing 5 mM Tris base, 15 mM NaCl and 0.1% (vol/vol) Triton X-100 (at pH 10) for 15 min at 60°C on the rocking shelves of the hybridization oven. After this washing step, the slides were rinsed again with ddH_2_0 and air-dried in the dark at room temperature. Subsequently, the slides were counterstained with 6-diamidine-2-phenylindole dihydrochloride (DAPI) (Serva, Heidelberg, Germany), a DNA-intercalating agent that stains the chromosomes of both prokaryotic and eukaryotic cells, for 5 minutes at room temperature in the dark and rinsed with ddH_2_0. Before imaging, the slides were air-dried at room temperature in the dark.

### Assessment of reproducibility of FISH

The inter-run repeatability of FISH was evaluated by comparing the outcomes of two independent FISH runs for a subset of the samples. The second hybridization was performed 6 months later on a subset of 15% of the samples (N = 17), which reflects one FISH run. We selected 17 samples showing variable results for the species-specific signal and positive for the broad-range probe. A new hybridization spot was drawn and fresh hybridization and washing buffer was used on the exact same slides used in the first FISH run. Visual inspection by confocal microscopy was performed by the same microscopist.

### Microscopic analysis of hybridized samples

**Fig 1:**
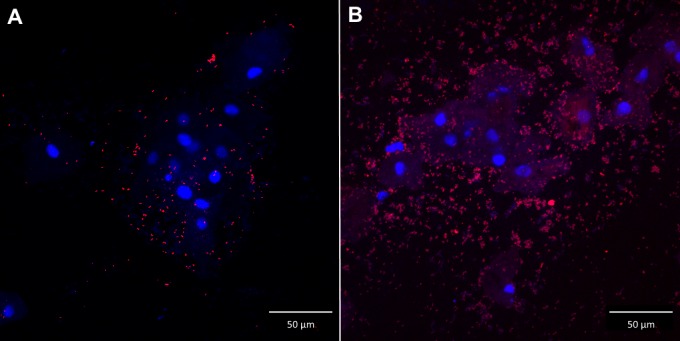
Dispersed bacteria versus biofilm. Confocal laser scanning images with 400x magnification of *G*. *vaginalis* biofilm in 2 vaginal slides (A and B) in a superimposed image: vaginal epithelial cells DAPI in blue and *G*. *vaginalis* specific PNA-probe Gard162 with Alexa Fluor 647 in red. A: vaginal sample with dispersed bacteria; B: vaginal sample with bacteria in biofilm.

The hybridized samples were stored in the dark at room temperature for a maximum of one week before microscopic observation using laser scanning confocal microscopy (LSM700, Zeiss, Oberkochen, Germany). The microscope operates with four stable, solid-state lasers at wavelengths of 405 to 639 nm, and is therefore able to detect all three fluorescently labelled probes and the DAPI stain at once in one hybridization chamber. The sample was first scanned at 100x magnification (objective: EC Plan-Neofluar 10x/0.30 Ph1 M27), before individual bacteria were identified at 400x magnification (objective: Plan-Apochromat 40x/1.3 Oil Ph3 M27). Separate scattered bacterial cells were defined as dispersed bacteria (Fig 1A). Aggregates of bacterial cells attached to the vaginal epithelial cells were defined as adherent bacteria forming a biofilm (Fig 1B). The species-specific signal was considered positive only if it had a positive counterpart in the DAPI stain and if it displayed a positive signal simultaneously with the broad-range probe.

### Statistical analysis

The specificity and sensitivity of FISH for vaginal slides was compared with the qPCR as a reference method. Bacterial counts were log 10 transformed before analysis. Data analysis was done using STATA13. The p-values reported for associations between the presence of bacterial species/biofilm and bacterial loads from the qPCR results originate from the non-parametric Kruskal-Wallis equality-of-populations rank test.

## Results

### PNA FISH probe performance on bacterial strains

The three probes specific for *A*. *vaginae* clearly hybridized with all eight *A*. *vaginae* strains tested (Table 2). However, Ato291 showed cross-hybridization with three *G*. *vaginalis* strains, with all *Lactobacillus* species and with one *Prevotella* strain. The newly developed AtoITM2 probe cross-hybridized with three *G*. *vaginalis* strains. Only AtoITM1 performed without false positive results and was selected for further evaluation on the clinical samples. The Gard162 probe was able to identify all five *G*. *vaginalis* test strains and showed no cross-hybridization with any of the other 15 species tested (Table 2). All bacterial strains tested hybridized with the broad-range BacUni-1 probe. According to the FISH results of the serial dilutions, the limit of detection for AtoITM1, Gard162 and BacUni-1 probes was 1.2 x 10^5^ cells per ml.

### Detection of *A*. *vaginae* and *G*. *vaginalis* in clinical samples by PNA FISH

In a small pilot study, a total of 10 paired vaginal slides and urine samples were tested to evaluate the suitability of each type of sample for FISH analysis. Six out of 10 urine samples could not be assessed due to the low presence of vaginal epithelial cells in the urine, whereas this problem was experienced in only 2 vaginal samples. Therefore, it was decided to continue analyses on vaginal slides only.

**Fig 2:**
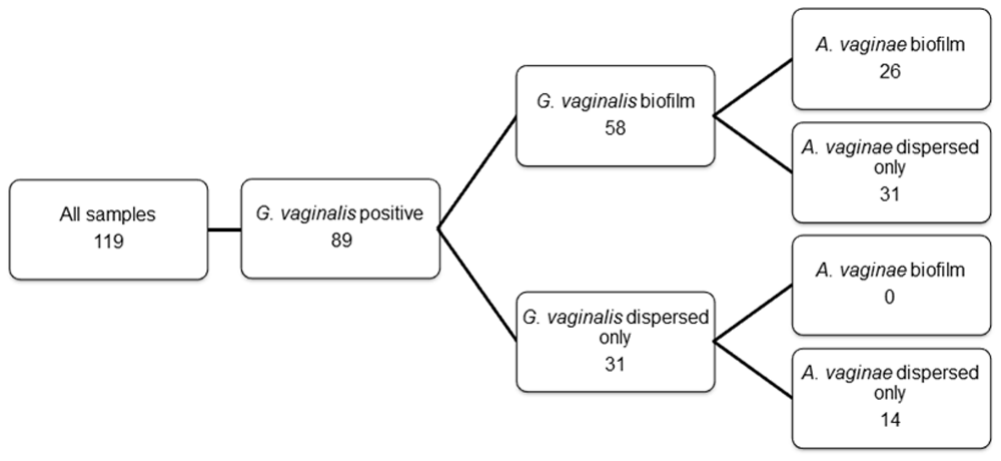
Distribution of samples according to FISH. Aggregates of bacterial cells attached to the vaginal epithelial cells, were defined as biofilm. Separate scattered bacterial cells, without the presence of biofilm, were defined as dispersed only bacteria.

**Fig 3:**
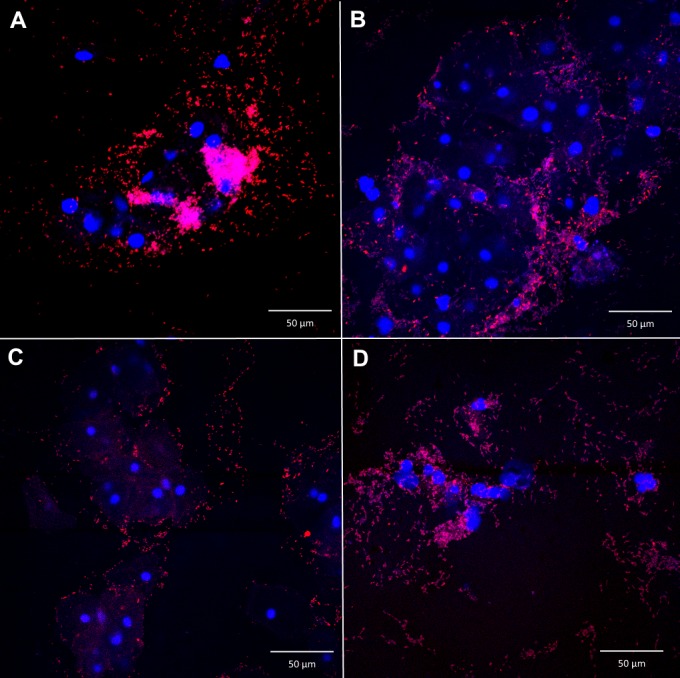
*G*. *vaginalis* biofilm. Montage of confocal laser scanning images with 400x magnification of *G*. *vaginalis* biofilm, negative for *A*. *vaginae*, in 4 vaginal samples (A-D) in a superimposed image: vaginal epithelial cells DAPI in blue and *G*. *vaginalis* specific PNA-probe Gard162 with Alexa Fluor 647 in red. For clarity we omitted the BacUni-1 plane; the bacteria that did not hybridize with Gard162 are visible in DAPI blue.

**Fig 4:**
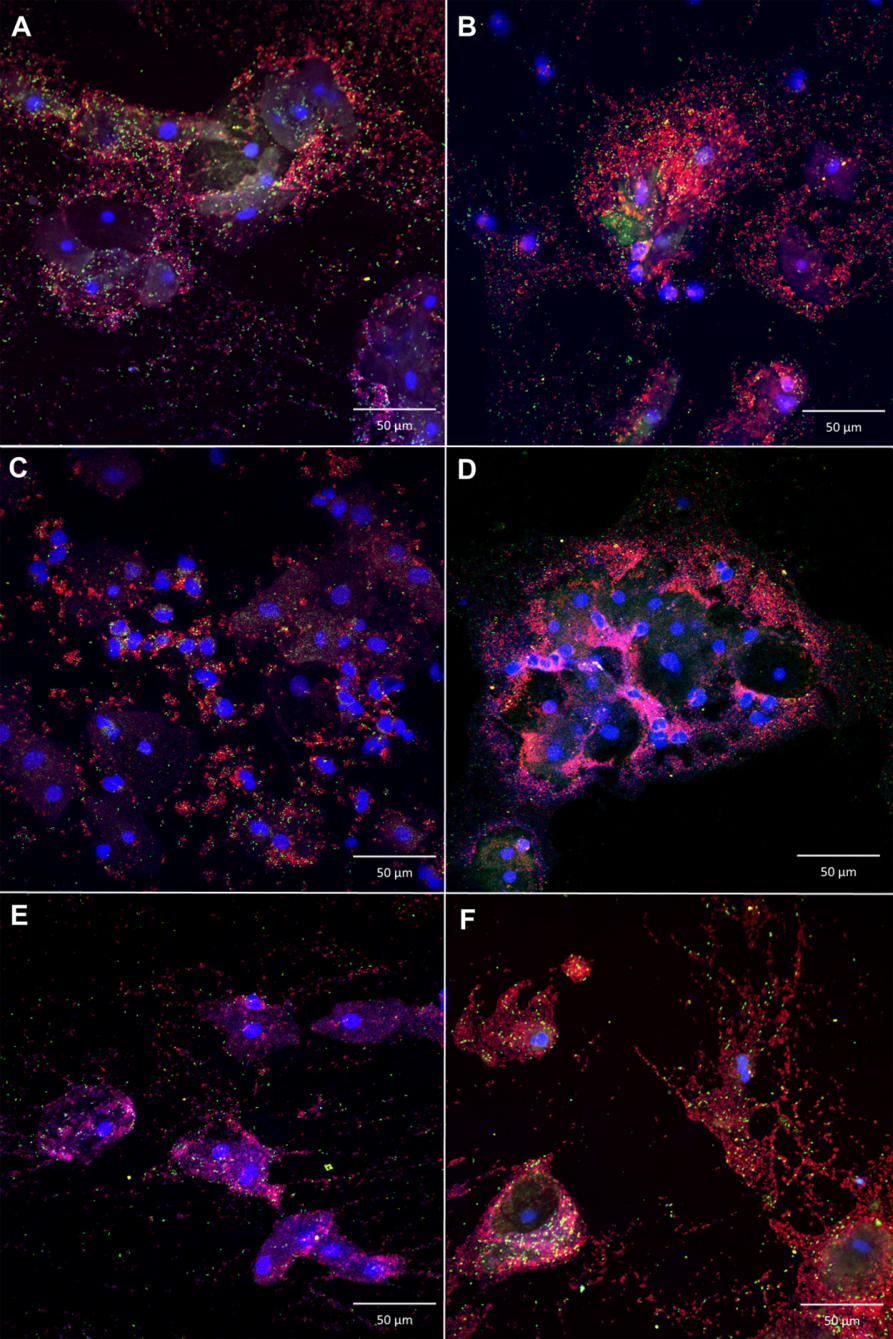
Superimposed image of polymicrobial biofilm of *A*. *vaginae* and *G*. *vaginalis*. Montage of confocal laser scanning images with 400x magnification of polymicrobial biofilm in 6 vaginal samples (A-F) in a superimposed image: vaginal epithelial cells DAPI in blue, *G*. *vaginalis* specific PNA-probe Gard162 with Alexa Fluor 647 in red and *A*. *vaginae* specific PNA-probe AtoITM1 with Alexa Fluor 488 in green. For clarity we omitted the BacUni-1 plane; the bacteria that are not bound by the specific probes are visible in DAPI blue.

**Fig 5:**
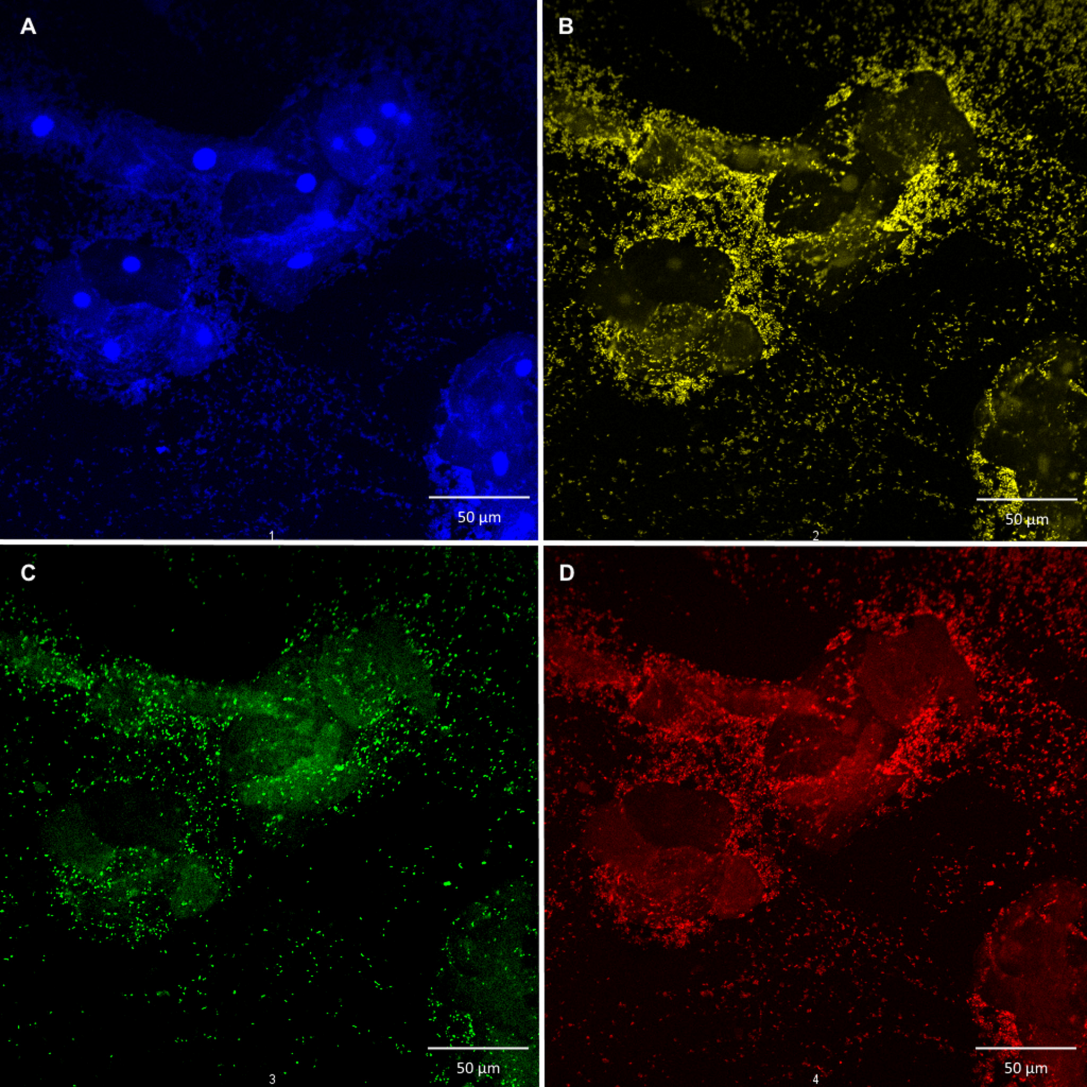
Polymicrobial biofilm of *A*. *vaginae* and *G*. *vaginalis* in different panes. Confocal laser scanning image with 400 x magnification of polymicrobial biofilm in different panes, A: vaginal epithelial cells DAPI in blue, B: all bacteria, BacUni-1 PNA-probe with Alexa Fluor 555 in yellow, C: *A*. *vaginae* specific PNA-probe AtoITM1 with Alexa Fluor 488 in green, D: *G*. *vaginalis* specific PNA-probe Gard162 with Alexa Fluor 647 in red (superimposed image can be seen in Fig 3A).

Using the AtoITM1 PNA-probe, *A*. *vaginae* was visualized as dispersed entities, without the presence of biofilm, in 27/119 (22.7%) of the samples. *A*. *vaginae* biofilm was present in 26/119 (21.8%) samples. *A*. *vaginae* FISH was negative in the remaining 66/119 (55.5%) samples. PNA-FISH using Gard162 detected dispersed-only *G*. *vaginalis* in 31/119 (26%) samples, *G*. *vaginalis* biofilm in 58/119 (48.7%) samples (e.g., Fig 2) and 30/119 (25.3%) samples were negative for *G*. *vaginalis*. Of the 89 *G*. *vaginalis* FISH-positive samples (dispersed or biofilm), 36 samples (41%) were negative for *A*. *vaginae*. However, all samples with *A*. *vaginae* biofilm showed a *G*. *vaginalis* biofilm as well (e.g., Figs 3, 4 and 5).

### Characterization of vaginal samples by qPCR

A total of 119 vaginal samples were available for qPCR analysis. *A*. *vaginae* was present in 72 (60%) of the samples with a mean log of 7.55 ± 1.34 geq/ml. *G*. *vaginalis* was detected in 95 (80%) of the samples with a mean log of 7.38 ± 1.11 geq/ml.

### Performance of probes in vaginal samples

Quantitative PCR was used as the reference method for detection and quantification of *A*. *vaginae* and *G*. *vaginalis*. The signal of the species-specific probes was only considered positive if a positive counterpart was seen in the DAPI stain and with the universal BacUni-1 probe. When assessing the results with FISH probe AtoITM1 against the qPCR outcomes for *A*. *vaginae* for 119 vaginal samples, FISH results were false negative for 24 samples and false positive for 5 samples, resulting in a sensitivity of 66.7% (95% confidence interval (CI): 54.5% - 77.1%) and a specificity of 89.4% (95% CI: 76.1% - 96%) (Table 3). The mean log for the true positive samples (positive with qPCR and FISH) was 7.73 geq/ml, as compared to a mean log of 7.19 geq/ml for the false negative FISH results (p = 0.399). For Gard162, the *G*. *vaginalis* probe, 13 FISH results were false negative and six false positive. The sensitivity was 86.3% (95% CI: 77.4% - 92.2%) and the specificity 75.0% (95% CI: 52.9% - 89.4%) (Table 3). The mean log for the true positive results for *G*. *vaginalis* was 7.61 geq/ml compared to a mean log of 5.94 geq/ml for the false negative results (p<0.001).

**Table 3:** Performance of *A*. *vaginae* (AtoITM1) and *G*. *vaginalis* (Gard162) PNA probes, compared to qPCR results, for 119 vaginal slides.

FISH	qPCR				
	*A*. *vaginae* positive	*A*. *vaginae* negative	*G*. *vaginalis* positive	*G*. *vaginalis* negative	Total
AtoITM1 positive	48 (66.7%)	5 (10.6%)			53
AtoITM1 negative	24 (33.3%)	42 (89.4%)			66
Gard162 positive			82 (86.3%)	6 (25%)	88
Gard162 negative			13 (13.7%)	18 (75%)	31
Total	72	47	95	24	

Assessment of the repeatability was done using 17 samples. After the first hybridization, all samples showed a signal for the BacUni-1 probe, 5 and 9 samples out of 17 for the AtoITM1 and Gard162 probe respectively. The results of the second FISH with the BacUni-1 and Gard162 probe were in full agreement with the first run. For the AtoITM1 probe, only one sample had a different result in the second run (negative at first, but positive in the second run).

### The presence of biofilm related to bacterial loads

The probability of detecting bacteria in a biofilm with FISH was higher when high (>10^6^ geq/ml) bacterial loads for *G*. *vaginalis* (p<0.001) and *A*. *vaginae* (p<0.001) were present. The mean log of both species was highest when *A*. *vaginae* was part of the biofilm, compared to a biofilm of *G*. *vaginalis* only. *A*. *vaginae* biofilm was always observed together with *G*. *vaginalis* (Table 4).

**Table 4:** Presence of *A*. *vaginae* and *G*. *vaginalis*, as assessed by FISH, in relation to *A*. *vaginae* and *G*. *vaginalis* loads as determined by qPCR for 119 vaginal samples.

	Total	*G*. *vaginalis* count 0	*G*. *vaginalis* count <10^6^ geq/ml	*G*. *vaginalis* count >10^6^ geq/ml		*A*. *vaginae* count 0	*A*. *vaginae* count <10^6^ geq/ml	*A*. *vaginae* count >10^6^ geq/ml	*A*. *vaginae* mean log
Detected by PNA FISH		N (%)	N (%)	N (%)	geq/ml	N (%)	N (%)	N (%)	geq/ml
***A*. *vaginae***									
Absent	66	20 (30.3%)	13 (19.7%)	33 (50.0%)	4.87	42 (63.64%)	7 (10.61%)	17 (25.76%)	2.55
Dispersed only	27	3 (11.1%)	0 (0.0%)	24 (88.9%)	6.82	3 (11.11%)	4 (14.81%)	20 (74.07%)	6.21
Biofilm^1^	26	1 (3.9%)	1 (11.8%)	24 (92.3%)	7.50	2 (7.69%)	0 (0%)	24 (92.31%)	7.66
***G*. *vaginalis***									
Absent	30	17 (56.7%)	6 (20.0%)	7 (23.3%)	2.57	24 (80.00%)	3 (10.00%)	3 (10.10%)	1.05
Dispersed only	31	2 (6.5%)	6 (19.4%)	23 (74.2%)	6.68	13 (41.94%)	5 (16.13%)	13 (41.94%)	3.97
Biofilm	58	5 (8.6%)	2 (3.5%)	51 (87.9%)	7.18	10 (17.24%)	3 (5.17%)	45 (77.59%)	6.55

^1^ *A*. *vaginae* biofilm = polymicrobial biofilm consisting of *A*. *vaginae* and *G*. *vaginalis*; No slides had *A*. *vaginae* biofilm only.

## Discussion

We set out to evaluate the performance of PNA FISH for the investigation of the vaginal polymicrobial biofilm consisting of *G*. *vaginalis* and *A*. *vaginae*. For this purpose, we evaluated three newly designed *A*. *vaginae* PNA probes for their specificity and applied the most specific one, AtoITM1, on a range of fixed vaginal slides together with an already existing *G*. *vaginalis* and broad-range PNA probe.

Hybridization-based techniques such as FISH have been used in various disciplines, such as cytogenetics and microbiology, to detect the presence or absence of nucleic acid sequences. Detection of DNA and RNA is generally done using DNA probes but the use of PNA probes is increasing. PNA molecules have a neutral backbone giving them a significant advantage in low ionic-strength conditions compared to DNA probes [40]. Low ionic-strength conditions prevent the complementary genomic sequences from reannealing when performing the FISH procedures; they facilitate denaturation of RNA secondary structures and favors hybridization of the PNA probes with nucleic acids. In combination with the superior penetration of PNA probes through the cell wall and hydrophobic bilayer of the target organism [41], PNA FISH is a fast, simple and robust assay. We compared DNA and PNA probes (data not reported) and can confirm that PNA FISH is faster and more robust than DNA FISH. In the current study, PNA FISH proved to be highly efficient for the identification and visualization of the spatial arrangement of *A*. *vaginae* and *G*. *vaginalis* in the BV-associated biofilms. Moreover, PNA FISH showed excellent inter-assay repeatability for all three probes used.

### PNA FISH probe performance on bacterial strains

For the design of the PNA probes, we opted for Alexa fluorochromes, which have similar spectral properties as other fluorochromes, such as cyanine dyes, but are brighter and more resistant to photo bleaching [42].

PNA probes that specifically target *A*. *vaginae* have not been described before. The only probe thus far reported was a DNA probe (Ato291) [27]. The probe was designed to detect bacteria belonging to the *Atopobium* cluster in fecal samples. However, *in silico* evaluation of the specificity of the Ato291 probe showed cross-hybridization with other bacteria belonging to the Coriobacteriaceae, a family of Actinobacteria, to which *A*. *vaginae* belongs. This is not surprising, since the probe was originally designed on the basis of sequences of Coriobacteriaceae strains isolated from feces and clinical material. The probe has been used for the detection of *A*. *vaginae* in vaginal samples by Swidsinski et al. [9], but their findings have not yet been confirmed by other groups. In our experiments, using a PNA equivalent of the Ato291 probe, we showed a low specificity of the Ato291 probe on vaginal clinical isolates. The Ato291 probe cross-hybridized with three out of five *G*. *vaginalis* strains and all five *Lactobacillus* species. Therefore, we designed two new probes for *A*. *vaginae* targeting the 16S rRNA-gene, based on published PCR primers [28,29] and we adjusted the sequences to fit the requirements for PNA probes. One of the new probes, AtoITM1, did not cross-react to any of the tested species and was further used for detection of *A*. *vaginae* in vaginal slides.

Gard162 is the first PNA FISH probe designed specifically for *G*. *vaginalis* and has extensively been tested by Machado et al. on a variety of cultured bacterial strains and clinical samples [30,31]. Using this probe, we obtained clear hybridization for all *G*. *vaginalis* isolates tested and observed no cross-reaction with strains of the other species tested, confirming the findings of Machado et al. [30].

### PNA FISH probe performance on clinical samples

Vaginal slides proved to be a valid sample type for imaging of the composition of the vaginal microbiome, if processed directly after sampling, as shown by Peltroche-Llacsahuanga et al. [43]. Collection of a vaginal swab is an easy and cheap sampling method, with a minimal burden on the study participant or patient. After heat fixation, we recorded that the slides can be stored at room temperature for up to at least six months and can be easily transported. A high quality vaginal sample can be obtained by thinly rolling the swab onto the slide. A thick vaginal ‘smear’ on the contrary where the material is smeared onto the slide is not useful for FISH and microscopic visualization.

The probes were also applied to 119 vaginal slides from women for whom the bacterial loads of *G*. *vaginalis* and *A*. *vaginae* had been quantified by qPCR. qPCR was used in this study as the reference method to evaluate the performance of the FISH probes, although comparison of these methods is subject to some limitations. qPCR is highly sensitive and was performed on a homogenized DNA extract representing half of the full sample. FISH however was carried out on 0.5 mm² of a vaginal slide, which could be heterogeneous. This is inevitably an underrepresentation of the vaginal sample. Both techniques were also performed using two different vaginal swabs, but the first collected specimen was used to prepare the slide as per study protocol.

After hybridization for 60 minutes and washing for 15 minutes, both at 60°C, the AtoITM1 probe gave only five false positive results, compared to qPCR results, resulting in a specificity of 90% for this set of samples However, 24 samples that were positive according to the qPCR were not detected by FISH, which gives a sensitivity of 67%. These results are comparable to the sensitivity observed for vaginal samples using FISH for detection of Group B *Streptococcus* [43]. The authors of this study obtained a higher sensitivity after extraction of the swabs by centrifugation of the swab head in a NaCl solution [43], but this method would probably disrupt the epithelial biofilm and thus prevent us from investigating the organization of the bacterial biofilm.

The relatively low sensitivity of the *A*. *vaginae* FISH assay cannot be explained by the bacterial load as measured by qPCR; the mean log of the true positive and false negative samples was not significantly different (log 7.73 and 7.19 geq/ml respectively). One possible explanation could be the typical structure of a biofilm, whereby an oxygen gradient exists from the top to the center of the biofilm [44]. Anaerobic bacteria such as *A*. *vaginae* are possibly found more embedded in the biofilm, to take advantage of the anaerobicity. It could be that the PNA probes are not able to fully penetrate into the inner parts of the biofilm; or that if the PNA probes do penetrate, the fluorescence could be masked and not detected due to low resolution of the equipment.

For *G*. *vaginalis*, 6 false positive results and 13 false negative results were found compared to 95 positive and 24 negative samples according to qPCR. This implies a sensitivity of 86% and specificity 75% for the detection of *G*. *vaginalis* by the Gard162 probe using our FISH protocol for this set of samples. This is lower than reported by Machado et al. [30], who, using the same probe, showed a full agreement between qPCR and FISH results for 13 vaginal samples. We were not able to elucidate why these discordant results were obtained.

### Bacterial loads and the presence of a biofilm

Our study shows that higher bacterial loads of *G*. *vaginalis* and *A*. *vaginae*, as detected by qPCR, are associated with a higher probability of presence of a bacterial biofilm. Both bacterial species are important constituents of the vaginal epithelial biofilm [9,45]. No samples contained *A*. *vaginae* in the absence of *G*. *vaginalis*, but almost half of the *G*. *vaginalis*-positive samples did not contain *A*. *vaginae* according to FISH results. Both bacteria were seen in a dispersed and an adherent state, but *A*. *vaginae* was always accompanied by *G*. *vaginalis*. The mere presence of *A*. *vaginae* did not simply predispose to a polymicrobial biofilm, but when *A*. *vaginae* was part of the biofilm, compared to a biofilm of only *G*. *vaginalis*, both bacterial species were present in higher concentrations.

We hypothesize that *G*. *vaginalis* is one of the main initiators of a vaginal biofilm, when it is present in high amounts. This vaginal biofilm creates a favorable environment for anaerobic bacteria, such as *A*. *vaginae*. One reason for the appearance of *A*. *vaginae* may be the presence of an oxygen gradient within the biofilm. By embedding itself within the biofilm, *A*. *vaginae* can take advantage of the anaerobicity, proliferates and exists in a mutualistic relationship with *G*. *vaginalis*.

## Conclusion

Our study confirms that PNA FISH is a valuable tool for detecting and visualizing biofilm-associated organisms in vaginal slides. This study describes the design and evaluation of a new PNA probe, AtoITM1, which can be included in multiplex FISH in BV biofilm research. Using the new probe, we have demonstrated the presence of a polymicrobial biofilm, with *A*. *vaginae* taking part in a *G*. *vaginalis* dominated biofilm.

## Supporting Information

S1 Protocol Final Approved Protocol for "The Ring Plus project: Safety and acceptability of vaginal rings that protect women from unintented pregenancy" version 2.0, 16 April 2013. (PDF)
