# Supplementary material for: Unravelling the Bacterial Vaginosis-Associated Biofilm: A Multiplex Gardnerella vaginalis and Atopobium vaginae Fluorescence In Situ Hybridization Assay Using Peptide Nucleic Acid Probes
Source: PLoS One. 2015 Aug 25;10(8):e0136658. doi: 10.1371/journal.pone.0136658 (PMC4548953; doi:10.1371/journal.pone.0136658)
Supplement: S1 Protocol — (PDF) [file pone.0136658.s001.pdf]

ITM Clinical Trial ITMC0313 / ClinicalTrials.Gov. NCT01796613

The Ring Plus Project: Safety and acceptability of vaginal rings that protect women from unintended pregnancy

Initial Protocol
Version 2.0, 16 April 2013

**Sponsor:**	Institute of Tropical Medicine<br>Nationalestraat 155<br>B-2000 Antwerpen - Belgium
**Coordinating Investigators:**	Tania Crucitti: sponsor coordinating investigator<br>Evelyne Kestelyn: country coordinating investigator

**Protocol Number:**	ITM C0313
**ClinTrialGov. Nr.:**	NCT01796613
**Title:**	The Ring Plus Project: Safety and acceptability of vaginal rings that protect women from unintended pregnancy
**Version:**	2.0
**Date:**	16 April 2013
**Previous version:**	1.0
**Sponsor:**	Institute of Tropical Medicine, Antwerp, Belgium
**Partnering institutions/sites:**	Rinda Ubuzima (RU), Kigali, Rwanda<br>HIV/STI Reference Laboratory, Clinical Trials Unit, and HIV/STI Epidemiology and Intervention Unit, Institute of Tropical Medicine, Antwerp, Belgium<br>Institute of Infection and Global Health, University of Liverpool, UK
**Study drugs:**	NuvaRing®: etonogestrel/ethinylestradiol
**Coordinating Investigators:**	Sponsor Coordinating Investigator<br>Dr. Tania Crucitti,<br>HIV/STI Reference Laboratory<br>Department of Clinical Sciences<br>Institute of Tropical Medicine, Nationalestraat 155, 2000 Antwerp, Belgium<br><br>Principal Investigator<br>Dr. Stephen Agaba<br>Rinda Ubuzima<br>KN 50th Av., Kiyovu, Kigali<br>Rwanda<br><br>Country Coordinating Investigator<br>Ms. Evelyne Kestelyn<br>Rinda Ubuzima<br>KN 50th Av., Kiyovu, Kigali<br>Rwanda
**Telephone sponsor:**	+32 3 247 65 52
**Fax sponsor:**	+32 3 247 66 47
**Email sponsor:**	tcrucitti@itg.be

PROTOCOL APPROVAL

SPONSOR COORDINATING INVESTIGATOR:

Title, Name: Dr. Tania CRUCITTI Date:
Signed:

DIRECTOR OF THE INSTITUTE OF TROPICAL MEDICINE:

Title, Name: Prof. Dr. Bruno GRYSEELS Date:
Signed:

COUNTRY COORDINATING INVESTIGATOR

Title, Name: Ms. Evelyne KESTELYN Date:
Signed:

STATEMENT OF COMPLIANCE & CONFIDENTIALITY

By signing this protocol, the Principal Investigator acknowledges and agrees:

This protocol contains the necessary information for conducting this clinical study. The Principal Investigator will conduct this study as detailed herein and will make every reasonable effort to complete the study within the time designated. The Principal Investigator commits to carry out the study in compliance with the protocol, amendments, applicable procedures and other study-related documents provided by the Sponsor, and in compliance with the most recent version of the Declaration of Helsinki, WHO and ICH Good Clinical Practice (GCP) and applicable regulatory requirements.

The protocol and all relevant study information, which is provided by the Sponsor, will be made available to the physicians, nurses, laboratory staff and other personnel who participate in conducting this study. The Investigator will use this material for their training so that they are fully informed regarding the drugs and the conduct of the study.

This document contains information that is privileged and confidential. As such, it may not be disclosed to any other persons than involved research staff and the concerned Ethics Committees, unless specific permission is granted in writing by the Institute of Tropical Medicine, Antwerp, Belgium, or such disclosure is required by federal or other laws or regulations. Additional disclosure guidelines are documented in the contract agreement and will be followed as agreed by the steering committee. In any event, persons to whom the information is disclosed must be informed that the information is confidential and may not be further disclosed by them. These restrictions on disclosure will apply equally to all future study-related information supplied which is regarded as privileged or confidential.

The Sponsor of this study – the Institute of Tropical Medicine in Antwerp, Belgium (ITM) – will at any time have access to the source documents from which Case Report Form information may have been generated. The Case Report Forms and any other data pertinent to this study are the property of the Sponsor. The data may only be utilized upon review and after discussion with the steering committee and/or the Sponsor. All study material will be maintained according to regulatory requirements and until the Sponsor advises that it is no longer necessary.

I, the Principal Investigator; agree to conduct the present study in full accordance with the most recent approved version of the protocol, within applicable timelines, according to the relevant standard operating procedures and in full agreement with all applicable regulations and the international guidelines regarding the conduct of clinical research.

I, the Principal Investigator; by signing this protocol declare that I will permit trial-related monitoring, audits, Independent Ethic Committee review, and regulatory inspections, providing direct access to source data/documents during and after the course of the trial.

I, the Principal Investigator; by signing this protocol declare that I will make the protocol and all relevant related information available to all physicians, nurses, laboratory staff and other personnel who participate in conducting this study. I will as well ensure that the study team at my site receive adequate training so that they are fully informed and qualified for the conduct of the study.

I also acknowledge the paragraph relevant to study confidentiality and authorize the Institute of Tropical Medicine, Antwerp, Belgium to record my data on a computerized system containing all the data pertinent to the study.

PRINCIPAL INVESTIGATOR:

Title, Name: Dr. Stephen Agaba ______________ Date: ______________
Signed:

TABLE OF CONTENTS

PROTOCOL APPROVAL 3
STATEMENT OF COMPLIANCE & CONFIDENTIALITY 4
TABLE OF CONTENTS 6
1. PROTOCOL TEAM ROSTER 8
1.1. THE INSTITUTE OF TROPICAL MEDICINE (ITM) 8
1.2. RINDA UBUZIMA 8
1.3. UNIVERSITY OF LIVERPOOL (UOL) 8
2. LIST OF ABBREVIATIONS 9
3. SYNOPSIS 11
4. INTRODUCTION 13
4.1. BACKGROUND 13
4.2. RATIONALE 14
5. STUDY DESIGN 15
5.1. AN INTEGRATED STUDY DESIGN 15
5.2. CLINICAL STUDY DESIGN 15
6. STUDY OBJECTIVES AND ENDPOINTS 16
7. SUBJECTS, POPULATION & SELECTION 18
7.1. SETTINGS, SELECTION & RECRUITMENT 18
7.2. INCLUSION AND EXCLUSION CRITERIA 18
7.3. SAMPLE SIZE 19
7.4. RANDOMIZATION AND BLINDING 20
7.5. WITHDRAWAL AND TERMINATION OF THE STUDY 20
8. STUDY PROCEDURES 21
8.1. STUDY/VISIT SCHEDULE 21
8.2. OBTAINING INFORMED CONSENT 26
8.3. SPECIFIC DATA COLLECTION PROCEDURES 26
8.4. LABORATORY PROCEDURES 28
9. STUDY INVESTIGATIONAL PRODUCT 29
9.1. PURCHASING, PREPARATION AND ADMINISTRATION 29
9.2. SUBJECT COMPLIANCE MONITORING 30
9.3. CONCOMITANT THERAPY 30
9.4. PACKAGING 30
9.5. RECEPTION, STORAGE, DISPENSING AND RETURN 30
10. SAFETY ASSESSMENT 30
10.1. ADVERSE EVENTS 30
10.2. DATA AND SAFETY MONITORING BOARD 33
11. ANALYSIS METHODS 33
11.1. CLINICAL DATA 33
11.2. QUALITATIVE DATA 36
12. MONITORING AND QUALITY ASSURANCE 37
12.1. LABORATORY QUALITY CONTROL AND QUALITY ASSURANCE 37
13. DATA MANAGEMENT 38
13.1. DATA MANAGEMENT - CLINICAL TRIAL 38
14. ETHICAL ISSUES 40

14.1. REGULATORY AUTHORITIES AND ETHICAL REVIEW 40
14.2. PROTOCOL AMENDMENTS 40
14.3. INFORMED CONSENT 41
14.4. CONFIDENTIALITY 41
14.5. RISKS AND BENEFITS 41
14.6. COMPENSATION FOR PARTICIPATION 42
14.7. INSURANCE 42

15. STEERING COMMITTEE AND TRIAL MANAGEMENT GROUP 43

16. ARCHIVING 43

17. REFERENCES 44

18. ANNEXES 45

ANNEX 1: CONTACT DETAILS OF ALL COLLABORATORS 45
ANNEX 2: WORLD MEDICAL ASSOCIATION DECLARATION OF HELSINKI 46

1. PROTOCOL TEAM ROSTER

1.1. The Institute of Tropical Medicine (ITM)

Address: Nationalestraat 155, 2000 Antwerpen, Belgium

Tania Crucitti, PharmD, Clin Biol, PhD
Sponsor Coordinating Investigator
Head of STI Reference Laboratory
HIV/STI Reference Laboratory
Department of Clinical Science

Thérèse Delvaux, MD, PhD
Leader Social Science Package
HIV/STI Epidemiology and Intervention Unit
Department of Public Health

Vicky Jespers, MD, MSc Epidemiology
Group Leader Biomarker and Microbicides
HIV/STI Epidemiology and Intervention Unit
Department of Public Health

Irith De Baetselier, MSc
Study Coordinator
Jr. Scientist
HIV/STI Reference Laboratory
Department of Clinical Science

Clinical Trials Unit (CTU) of ITM
Department of Clinical Science

1.2. Rinda Ubuzima

Address: KN 50th Av. 716, Kiyovu, Kigali, Rwanda

Stephen Agaba, MD
Principal Investigator

Jean-Claude Ndagijimana, MD
Study Physician and Co-Investigator

Evelyne Kestelyn, MA, MPH
Country Coordinating Investigator
Director of RU & pre-doctoral social scientist

Mireille Uwineza
Study Coordinator

1.3. University of Liverpool (UoL)

Address: Ronald Ross Building, 8 West Derby Street, Liverpool, UK

Janneke van de Wijgert, PhD, MPH, MSc
Professor UoL Institute of Infection and Global Health
Chair of the RU Board

Jennifer Ilo Van Nuil, PhD
Post-doctoral social scientist located at RU
UoL Institute of Infection and Global Health

A more detailed listing with the contact details of all collaborators and their responsibilities in the study can be found in Annex 1.

2. LIST OF ABBREVIATIONS

ACASI	Audio computer-assisted self-interviewing
BV	Bacterial vaginosis
CA	Competent Authorities
CDM	Clinical Data Management
CI	Coordinating Investigator
(e)CRF	(electronic) Case Report Form
CVL	Cervicovaginal lavage
CVR	Contraceptive Vaginal Ring
CT	*Chlamydia trachomatis*
CTU	Clinical Trials Unit
DSMB	Data and Safety Monitoring Board
EC	Ethics Committee
EDC	Electronic Data Capture
EDCTP	European & Developing Countries Clinical Trials Partnership
FDA	Food & Drug Administration
FGD	Focus Group Discussion
FISH	Fluorescence In Situ Microscopy
FP	Family Planning
GCP	Good Clinical Practice
GCLP	Good Clinical Laboratory Practice
HIV	Human Immunodeficiency Virus
HSV	Herpes simplex virus
IAQ	Interviewer Administered Questionnaire
IC	Informed Consent
ICH	International Conference on Harmonization
IDI	In-depth Interview
IL	Interleukin
IP	Investigational Product
IPM	International Partnership of Microbicides
IRB	Institutional Review Board
ITM	Institute of Tropical Medicine
MTN	Microbicide Trials Network
NG	*Neisseria gonorrhoea*
PCA	principal component analysis
PCR	Polymerase Chain Reaction
PI	Principal Investigator
PSA	Prostate specific antigen
QA	Quality Assurance
QC	Quality Control
qPCR	Quantitative real time polymerase chain reaction
RU	Rinda Ubuzima
(S)AE	(Serious) Adverse Event
SC	Steering Committee
SOP	Standard Operating Procedure
STI	Sexually Transmitted Infection
TMG	Trial Management Group
TV	*Trichomonas vaginalis*

UoL	University of Liverpool
US	United States
UZA	Universitair Ziekenhuis Antwerpen (~ University Hospital Antwerp)
VIA	Visual Inspection with Acetic acid
WHO	World Health Organization

3. SYNOPSIS

Purpose	The study is a multidisciplinary research project and has two main aims:<br>1) To determine the safety of a contraceptive vaginal ring (CVR) in women, with an emphasis on its effect on the vaginal microenvironment after different durations of use: the vaginal microbiome, biofilm formation on epithelial cells and rings, inflammation and immune activation in the vagina<br>2) To investigate the feasibility, acceptability and adherence to vaginal ring use in Rwandan women, including attitudes towards a future multi-purpose vaginal ring for prevention of both pregnancy and sexually transmitted infections (STI).
Study Design	The first component is an open label, single centre, randomized controlled trial. A total of 120 HIV-negative women will be randomized to an intermittent regimen (3 weeks ring use followed by one week of no ring use to allow menstruation) or a continuous regimen (3 weeks of ring use with no off period. The next ring is immediately inserted after the previous one). Women will be followed for maximum 14 weeks.<br>The second component is a qualitative study using in-depth interview (IDI) and focus group discussion (FGD) methodology.
Study site	Rinda Ubuzima, Kigali, Rwanda
Study Product	NuvaRing®: etonogestrel/ethinylestradiol
Study Population	HIV negative, healthy women who are currently not using a modern contraceptive method (not including barrier methods) and who are interested in initiating hormonal contraceptive use.
Duration	The individual participant will start CVR use at the enrollment visit after having given written informed consent and having received baseline examinations. Women will be followed-up 3 or 4 times (depending on ring use regimen) at the study clinic while using the ring and after completion of 3 months ring use. The participant will be enrolled within 6 weeks of the start of the screening process. The total study duration per participant will be a maximum of 5 months. We expect the total study duration of the entire study to be around 7 months.
Objectives of the Clinical Trial	*Primary Objective:* To assess the impact on the vaginal microbiome of the use of a vaginal ring intermittently or continuously.<br>*Secondary Objectives:*<br>1. To assess the general safety of the CVR;<br>2. To assess vaginal biofilm formation and to detect the presence or absence of a biofilm on the CVRs after intermittent or continuous use;<br>3. To determine the impact of intermittent or continuous use of the CVR on markers of inflammation and immune activation in the vagina.

Objectives of the Social Science Component	_Primary Objectives:_ To assess the level of acceptability and reported adherence to intermittent and continuous CVR use in women in Rwanda.<br>_Secondary Objectives:_ To identify and describe the context specific attitudes and beliefs regarding family, family planning, sexuality, and gendered norms.<br>_Exploratory Objectives:_ To explore how women and men in Rwanda perceive and experience risk related to unwanted pregnancy and HIV and their attitudes and expectations toward multi-purpose rings (e.g. HIV and family planning).
Endpoints of Clinical Trial	_Primary Endpoints:_ changes from baseline (pre-ring use) in vaginal bacterial counts and in the absence/presence of Bacterial vaginosis (BV)-related bacteria as measured with quantitative real time PCR.<br>_Secondary Endpoints:_<br>1. Incidence of self-reported vaginal symptoms and clinician-observed signs;<br>2. Incidence of BV;<br>3. The numbers of women prematurely withdrawing from the study or discontinuing ring use;<br>4. Incidence of adverse and serious adverse experiences;<br>5. The numbers of incident STIs and candidiasis;<br>6. Nugent scores and Ison & Hay grading of the vaginal flora;<br>7. Changes in phenotype of BV-related bacteria;<br>8. Presence or absence of a biofilm on the vaginal rings;<br>9. Changes in inflammatory cytokines concentrations in CVLs as measured by Luminex technology.
Endpoints of the Social Science Component	_Primary Endpoints:_ Lost to follow up rate; rate of discontinuation; level of adherence, qualitative description of user experiences, relationship attributes, and sexual encounter attributes; reported vaginal practices and beliefs regarding menstruation; reported reproductive health symptoms and number of women envisaging future use of CVR.<br>_Secondary Endpoints_: Qualitative description regarding women's views on family structure, family planning methods, sexuality, and community norms and perceptions regarding family planning (FP) and sexuality.<br>_Exploratory Endpoints_: Qualitative descriptions of beliefs, attitudes, and expectations regarding multi-purpose rings; number of women reporting likelihood of future use of multi-purpose ring; qualitative description of perceived risk of HIV and unwanted pregnancy and personal experiences of risky situations.

4. INTRODUCTION

4.1. Background

Vaginal rings are polymeric drug delivery devices designed to provide controlled release of drugs for intravaginal administration over extended periods of time. Compared to systemic dosing, the sustained local release of drug maximizes efficacy at lower doses, thereby reducing side effects [1-3]. Furthermore, the ring can stay in place for 1-3 months, which improves adherence [4-6]. In recent years, vaginal rings have become popular for contraception and estrogen replacement therapy in Latin America, the US and Europe where studies on the acceptability of the CVR have shown overall good adherence and acceptability. On average, 85% of users were satisfied or very satisfied and 85% to 90% would recommend the method to others [3;7].
However, CVRs are not yet on the market in any African country, and acceptability of such rings has not yet been studied. Limited vaginal ring acceptability data are available in the context of HIV prevention research. A study by the International Partnership for Microbicides (IPM) in Tanzania and South Africa evaluated the acceptability of a silicone elastomer vaginal ring not containing any active drugs (placebo ring). The study found that the vast majority of women felt comfortable inserting and removing the ring, acceptability increased with prolonged use, and only one male sexual partner felt the ring during sex [8]. This placebo ring, and a similar ring containing the antiretroviral drug dapivirine, is currently used in two HIV prevention efficacy trials in African women (the IPM027 and MTN020 trials) [1]. IPM and CONRAD have elaborated plans to develop multi-purpose vaginal rings that would be used for contraception as well as prevention of transmission of STIs, including HIV infection. Given these developments, we believe that single-purpose and/or multi-purpose vaginal rings will eventually be introduced in African settings.
A multi-purpose vaginal ring will have to be used continuously, which is different from the way CVRs currently are mostly used. Current use is intermittent, i.e. the ring is taken out for 1 week after having been in place for 3 weeks. We need in-depth data on the acceptability of the vaginal ring and on the acceptability of continuous use as women will not have a regular menstrual cycle anymore. Gaining a better understanding of acceptability requires the assessment of an intertwined set of variables and constructs such as users' personal characteristics and factors influencing the user experience (product efficacy, attributes, and price; relationship context and factors impacting on sexuality). These factors in turn are embedded in a specific sociocultural context (e.g. gendered sexual norms and culturally grounded beliefs about contraceptives). The conceptual framework developed by Woodsong et al. for assessing the vaginal ring for microbicide delivery will guide our acceptability research [9].

BV is an imbalance of the vaginal microbiome (a 'microbiome' is a community of different types of bacteria that live on surfaces and in the human body, such as the skin, mouth, gut and vagina). Epidemiological studies have shown that women with BV (defined as a Nugent score of 7-10 when reading a Gram-stained microscopy slide) are twice more likely to acquire HIV than women without BV [10, 11]. The vaginal microbiome can now be characterized in much more detail using molecular methods than was previously possible using microscopy. Very recent research has shown that the vaginal microbiome in African women can be categorized into at least 6 different bacterial communities, and that each bacterial community has distinct characteristics in terms of the types and quantities of bacteria present, whether or not inflammation is induced, and whether there is an increased risk for HIV or other STI

(unpublished data; publications in progress). The presence of inflammation is particularly important because inflammation often causes symptoms (itching, burning) and is associated with an influx of CD4+ T cells and macrophages into the genital area (these are the cells that are the target cells for HIV). It is therefore very important that any product that is used inside the vagina (such as a CVR) does not cause irritation and/or inflammation. The role of hormones in inflammation is also not yet clear. The presence of inflammation in the vagina can be measured in vaginal fluids (cervicovaginal lavages or CVLs) in multiple ways, such as measuring cytokines and chemokines directly, measuring mRNA that will be translated by the body into cytokines/chemokines, or measuring other proteins that are involved in the very complex pathways that are induced by cytokines/chemokines. At this point in time, it is not yet known which measures of inflammation are the most appropriate to determine product safety, which is why multiple measurements are often used.

BV has also been associated with biofilm formation of microorganisms on the vaginal epithelia. A biofilm is a complex aggregation of micro-organisms that excrete an adhesive and protective matrix and this may be the reason why BV recurrence rates are very high after initial successful treatment [12, 13]. BV is more common in African and African-American women than in Caucasian women, which could be due to the high prevalence of vaginal anaerobic bacteria that are prone to form biofilms [14, 15]. It is also well known that biofilms easily form on the surface of catheters and prostheses, such as artificial heart valves [16]. Knowledge is lacking on whether bacteria from the vaginal environment can adhere to the ring and form a biofilm. A small study among four non-human primates suggested that the vaginal ring could lead to biofilm formation in the animals [17]. We therefore need to explore the formation of biofilms on vaginal rings in the vagina for different periods of time. Furthermore, biofilms may hamper delivery of the active compounds contained within the rings and could lead to clinical BV (which in turn increases risk of HIV acquisition). Better knowledge on biofilm formation in the cervicovaginal context will aid further development of multi-purpose vaginal rings.

4.2. Rationale

Contraceptive and estrogen replacement therapy vaginal rings are already used in some countries, dapivirine vaginal ring efficacy trials are ongoing, and plans to develop multi-purpose vaginal rings have already been elaborated [18]. A multi-purpose vaginal ring will have to be used continuously, which is different from the way CVRs are currently mostly used. Current use is intermittent, i.e. the ring is taken out for one week after having been in place for 3 weeks. We need in-depth data on the safety and acceptability of the CVR and of the acceptability of continuous use as women will not have a regular menstrual cycle anymore. We plan to focus on safety aspects of CVR use that have not been studied in depth in the past, but are important in the African context, namely biofilm formation and induction of inflammation.
A second urgency is the unmet need for contraception in Africa, especially in Rwanda [19-21]. Several large epidemiological studies have suggested an increased risk of HIV acquisition in women using high dose long-acting progestogens (the so-called ‘injectables’) [22], which means that these may have to be replaced with other long-acting hormonal methods such as CVRs. However, this is of course only possible if those new hormonal methods do not pose an HIV acquisition risk by causing inflammation.

The CVR that will be used in this trial is commercialized under the trade name of NuvaRing®. It is approved since 2001 by most countries within the European Union, and in the United

Stated by the FDA. To date NuvaRing® is available in 32 countries and estimated to be used by 1.5 million women worldwide. NuvaRing® contains etonogestrel/ethinyl estradiol, it is manufactured by N.V. Organon (a subsidiary of Merck & Co., Inc.,), Oss, the Netherlands.

5. STUDY DESIGN

5.1. An integrated study design

This clinical study combines a clinical safety evaluation of the CVR with social science research on acceptability and adherence of ring use in Rwandan women.
The *clinical part* of the study assesses the general safety of the contraceptive ring including the effect on the vaginal microbiome, genitourinary symptoms and signs and clinical adverse events. In addition the *clinical part* of the study aims to understand better the possible biofilm formation and to explore the impact of the ring on the microbiome and inflammation in the vagina.
The *social science component* consists of a combination of qualitative and quantitative research including IDIs, FGDs, questionnaires, and (if funding permits) audio computer-assisted self-interviewing (ACASI).

5.2. Clinical study design

This is an open label single-centre cohort study with the NuvaRing®. A total of 120 HIV-negative women will be randomized to an intermittent regimen or a continuous regimen of ring use and will be followed for maximum 14 weeks, to determine general safety of the ring in the African context, and to determine differences in the vaginal microbiome and vaginal inflammation before and after use of a CVR. Qualitative research for the social science component will be performed to address the acceptability and adherence of intermittent or continuous CVR use in more depth.

Women will be randomized into two study groups: 60 intermittent users in group A and 60 continuous users in group B. Both groups will use the NuvaRing®. Women in group A (the intermittent users) will use each CVR for 3 weeks followed by one week of no ring use to allow menstruation. Women in group B (the continuous users) will use each ring for 3 weeks, with the next ring being inserted immediately after the previous one and no off period in between [4]. The total duration from enrolment to the last study visit is maximum 14 weeks. The screening process will be a maximum of 6 weeks.

Schedule

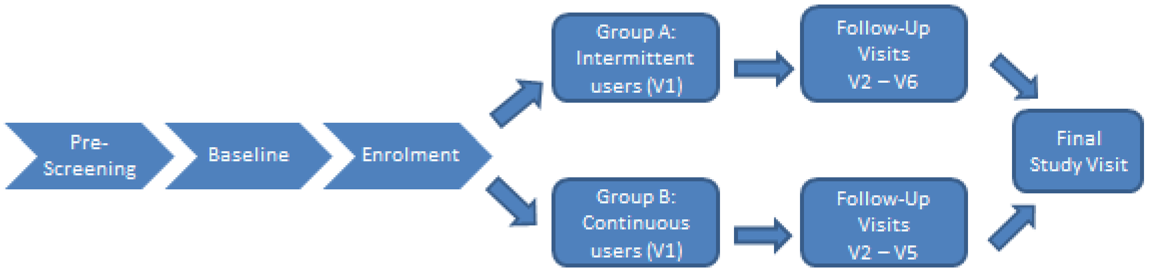

6. STUDY OBJECTIVES AND ENDPOINTS

**Study Method**	**Primary objectives**	**Primary endpoints**
Clinical Trial	To assess the impact on the vaginal microbiome of the use of a CVR intermittently (3 weeks followed by 1 week off) or continuously.	Changes from baseline (pre-ring use) in vaginal bacterial counts and in the absence/presence of BV-related bacteria as measured with quantitative real time PCR.
Social Science Component	To assess the level of acceptability and reported adherence to intermittent and continuous CVR use in women in Rwanda.	Lost to follow up rate; rate of discontinuation (base on clinical trial data).<br>Level of adherence based on self-reported adherence scale and diary cards.<br>Qualitative description of user experiences, relationship attributes, and sexual encounter attributes.<br>Reported vaginal practices and beliefs regarding menstruation and impact on ring adherence.<br>Reported reproductive health symptoms and impact on adherence.<br>Number of women envisaging future use of contraceptive ring.
**Study Method**	**Secondary objectives**	**Secondary endpoints**
Clinical Trial	To assess the general safety of the CVR.	Incidence of self-reported vaginal symptoms and clinician-observed signs<br>Incidence of BV.<br>The numbers of women prematurely withdrawing from the study or discontinuing ring use.<br>Incidence of adverse and serious adverse experiences .<br>The numbers of incident STIs and candidiasis.<br>Nugent scores and Ison & Hay grading of the vaginal flora.
Clinical Trial	To assess the vaginal biofilm formation according to ring use regimen.	Changes in phenotype of BV-related bacteria, specifically:<br>- Presence of dispersed forms of bacteria;<br>- Presence of adhesive forms of bacteria on epithelial cells.
Clinical Trial	To determine the presence or absence of a biofilm on the CVRs after intermittent or continuous use by visualization of the biofilm.	Presence or absence of a biofilm on the CVRs after intermittent or continuous use:<br>- Presence of biofilm visualized by crystal violet;<br>Visualization of adherent bacteria using FISH;<br>- Identification of adherent bacteria (including but not limited to *Lactobacilli sp.*, *G. vaginalis*, *A. vaginae*).

Study Method	Secondary objectives (cont.)	Secondary endpoints (cont.)
Clinical Trial	To assess the impact of CVR on vaginal inflammation and immune activation according to ring use regimen.	Changes in inflammatory cytokines concentrations in CVLs as measured by Luminex technology. The cytokines analyzed include, but are not limited to IL-1, IL-6, IL-8, IL-10. All samples that are showing elevated cytokine levels, as well as an equal number of controls not showing elevated cytokine levels, will be tested further for the presence of proteins involved in inflammatory pathways.
Social Science Component	To identify and describe the context specific attitudes and beliefs regarding family, FP, and gendered norms.	Qualitative description regarding women's views on family structure, family planning methods, sexuality, and community norms and perceptions regarding FP and sexuality.
**Study Method**	**Exploratory Objectives**	**Exploratory endpoints**
Social Science Component	To explore the women's beliefs and expectations regarding future potential use of a multi-purpose ring (contraception and HIV prevention).	Qualitative descriptions of beliefs, attitudes, and expectations regarding multi -purpose rings.<br>Number of women reporting likelihood of future use of multi-purpose ring.
Social Science Component	To explore how women and men in Rwanda perceive and experience risk related to unwanted pregnancy and HIV.	Qualitative description of perceived risk of HIV and unwanted pregnancy and personal experiences of risky situations.

7. SUBJECTS, POPULATION & SELECTION

7.1. Settings, selection & recruitment

A total of 120 HIV-negative women will be recruited from the general population. They will receive extensive counselling on all contraceptive methods that are available in Rwanda. Women will also be counselled on prevention of HIV and other STIs, and will be offered HIV and STI testing and condoms free of charge. The social science component of the acceptability study will include IDIs and FDGs and these will take place alongside the clinical study.

All participants will be recruited and followed up at the Rinda Ubuzima (RU) research clinic and laboratory in Kigali, Rwanda. Rinda Ubuzima (formerly Projet Ubuzima) was established in 2004 and has successfully conducted 7 clinical studies in the areas of HIV prevention and reproductive health between 2004 and 2012, including two clinical trials with an experimental vaginal microbicide. Rinda Ubuzima receives technical assistance in all of its studies from the University of Liverpool (UoL), Institute of Infection and Global Health.

The following sections describe the groups and the inclusion and exclusion criteria in detail.

7.2. Inclusion and exclusion criteria

In order to be eligible, study participants **must meet the following criteria**:

- Able and willing to give informed consent/assent, according to national guidelines
- Female who self-reports to be sexually active (meaning at least one penetrative vaginal coital act per month for the last 3 months prior to screening)
- Between 18 to 35 years old, inclusive
- Currently in good physical and mental health
- Interested in initiating hormonal contraception
- Able and willing to participate in the study as required by the protocol, this includes willing to undergo HIV testing and use a NuvaRing®
- HIV negative at screening as confirmed by rapid HIV testing

Potential participants meeting any of the following criteria **will not be enrolled in the study**:

- Currently using a modern contraceptive method other than barrier methods
- Use of a hormonal contraceptive method in the three months prior to the screening visit
- Currently using antimicrobial medication
- Pregnant on urine pregnancy test
- History of cardiovascular disease

- History of hysterectomy or genital tract surgery (including cervical polypectomy, dilatation and curettage, hysteroscopy, or laparoscopy) in the three months prior to the screening visit
- History of complications with hormonal contraception or with contra indications for the use of hormonal contraceptives such as:
    - History or known predisposition for venous thrombosis
    - History of migraine with focal neurological symptoms
    - Diabetes mellitus with vascular involvement
    - History of pancreatitis or severe hepatic disease
    - Known or suspected hypersensitivity to any of the excipients of NuvaRing®
- History of significant urogenital or uterine prolapse, undiagnosed vaginal bleeding, incontinence or urge incontinence, diagnosed chronic and/or recurrent vulvovaginal candidiasis, urethral obstruction
- Participating in other clinical studies involving investigational products
- Currently breastfeeding
- Currently a smoker

7.3. Sample size

A total of 120 women will be enrolled in the clinical trial: 60 women will be randomized to the intermittent regimen of ring use and 60 women to the continuous regimen of ring use. This will ensure we have 104 women with evaluable primary outcome data to provide sufficient power for the primary clinical trial objective .This is based on the sample size calculations presented in protocol section 11.1.3.

As is always the case in qualitative research, sample sizes are determined by when data saturation is reached (meaning: additional IDIs or FGDs do not generate new information that was not already mentioned in previous IDIs or FGDs). In this study protocol, we list estimates (based on our experience with previous qualitative research) of how many IDIs or FGDs are needed to reach data saturation, but fewer or more IDIs or FGDs may be done based on the actual results when we are implementing the study.

After pre-screening, IDIs will be conducted until data saturation is reached, which we assume to be the case after interviewing 10-15 women. Midway through the project, two FGD per randomization group will be conducted including 8-12 women per FGD (for a total of 32 to 48 women in 4 FGDs). At the end of the trial, one FGD (with 8-12 women per group) and 3-5 IDIs per randomization group will be conducted (for a total of 16-24 women and 6-10 women, respectively). Women participating in the end of trial FGDs and IDIs will be invited to ask their male partners to attend separate IDIs and 3-5 male partners will be interviewed in each randomization group (for a total of maximum 10 men). Additional FGD will be conducted with women who are not enrolled in the clinical study (8-12 women per FGD) until data saturation is reached: at least one FGD with women who attend a family planning clinic and at least one FGD with women who are HIV-positive.

7.4. Randomization and blinding

This is an open-label study because the differences in ring use (intermittent or continuous use) cannot be blinded. Individual participants will be randomized and treatment allocation will be concealed until a participant has provided informed consent, confirmed eligible, and is included in the study. Randomization will be done at enrolment using randomization envelopes, specifying the treatment group.

7.5. Withdrawal and termination of the study

Reasons for Withdrawal
All participants will be encouraged to continue in the study. A participant may voluntarily discontinue from the study at any point.

Participants may be withdrawn from the study if:

1. the participant or legal representative withdraws the consent;
2. the investigator considers that further participation would have negative effects on the woman's health;
3. the participant became pregnant.

Participants who are discontinued from the study will not be re-screened.

Handling of Withdrawals
Reasons for discontinuation from the study, date and time will be documented in the patient file and recorded on the study Case Report Form (CRF). When early discontinuation from the study due to safety reasons occurs, every reasonable effort will be made to conduct all procedures or at a minimum, participants will be seen for a final evaluation and will be followed up for safety reasons. When a participant becomes pregnant during the study, the participant will be followed up until the conclusion of the pregnancy and the outcome of the pregnancy will be documented in the patient file and reported on a study specific form.
When a participant or legal representative withdraws the consent, no additional procedures will take place.

In this study, a participant will be considered lost to follow-up at study closure if she discontinued study visits without informing the study staff and cannot be traced. Participants who miss visits but do return to the study clinic prior to study closure will not be considered lost to follow-up but as having discontinued CVR use. Participants who inform the study staff that they are terminating their participation in the study will be considered early withdrawals. Contact attempt and procedures will be conducted according to the study specific RU SOP for Study Participant Retention.

Termination of Study
If the study is prematurely terminated or suspended, the Sponsor will promptly inform the investigators/institutions involved in the study, the ethics committees, and the Rwanda Ministry of Health of the termination or suspension and the reason(s) for the termination or suspension. The Rwanda-based PI is responsible for informing active study participants of study termination or suspension.

8. STUDY PROCEDURES

8.1. Study/visit schedule

Also refer to schedule of assessments 8.1.8.

8.1.1. Screening

The screening process is in two parts and can take up to six weeks.

8.1.1.1. Pre-screening visit

RU will contact participants from previous studies who were willing and interested to participate in other studies. These women have previously consented to being contacted by RU staff, have indicated their preferred method of contact (in person at home or at work, by phone, etc.), and have provided their contact information. Standard RU procedures will be used to ensure that confidentiality is not breached. For example, the project vehicles do not carry a logo, outreach staff do not wear uniforms, and no information about the study is given to anyone other than the potential participant herself. If the target number is not reached by using this pool of potential participants, women from the general population will be recruited. Recruitment will be done in several catchment areas around the research site by collaborating with the local authorities and setting up information sessions to inform potential participants about the study. Potential participants who are interested in initiating hormonal contraception will be invited for a pre-screening visit at the RU Research Clinic.
Upon arriving at the RU Research Clinic, all potential eligible participants will be assigned a participant identification number. The informed consent (IC) process will be done according to the existing GCP-compliant RU IC SOP and will include a group educational session with a video screening, followed by an individual session, consisting of a formal assessment of literacy, a discussion with a nurse counselor and a formal assessment of understanding of the study including the IC procedures.
After written informed consent is obtained by the woman and, in case of women minor of age (<21) and not married by the parent or legal guardian (see section 8.2 for more details), staff will:

- Collect contact information
- Collect menses information.
- Conduct HIV and other STI counseling.
- All consenting, eligible women will be invited for a baseline visit, 4 to 6 weeks after the pre-screening visit. Women who are menstruating at the baseline visit will be rescheduled.

In addition:

- All women included in the clinical trial will be asked at pre-screening whether they agree to possibly participate in a qualitative component of the study i.e. an IDI or a FGD.
- A total of 5 women will be purposively selected initially, and if data saturation is not reached after these 5 interviews, additional women will be selected one by one until data saturation is reached but will not exceed 15 women. Each IDI participant will be asked to provide written informed consent for participation in the IDI. After consent has been given, the participant will be interviewed by a study staff member. The IDIs will help to refine the Interviewer Administered Questionnaires (IAQ) that will be administered to all clinical trial participants at baseline and follow-up visits.

8.1.1.2. Baseline visit (week 0)

First, women will be interviewed face-to-face using an IAQ. The IAQ will include questions about demographic characteristics, sexual behaviour, medical history and reproductive history, current medication, menses information and acceptability of the CVR.

The following procedures will also be carried out:

- A pelvic examination:
    - A total of 3 vaginal swabs will be collected:
        - One cotton swab for Wet Mount [*Trichomonas vaginalis* (TV); *Candida albicans*] and smears for BV diagnosis;
        - Two regular flocked swabs (Copan, Italy) for the characterisation of the vaginal microbiome.
    - A total of 2 cervical swabs will be collected for *Neisseria gonorrhoeae* (NG) and *Chlamydia trachomatis* (CT) testing. One swab will be processed immediately for the rapid detection of CT. The other regular flocked swab will be used for molecular testing of CT/NG.
    - A CVL for the detection of inflammatory processes.
- A physical examination of other body systems will be performed with special attention to the cardiovascular system.
- Collect blood by venepuncture (5 ml) in a serum tube and test for HIV according to the national HIV algorithm, and for syphilis and herpes simplex type 2 (HSV-2).
- A urine sample will be obtained for testing for pregnancy and an aliquot of fixated urine for FISH analysis will be stored at 2-8°C until shipment to ITM.
- All women will be offered risk reduction counselling, including pre- and post-test HIV counselling and condoms free of charge.
- Curable genital infections will be treated at the study site free of charge.
- Any other medical conditions – including newly diagnosed HIV – will be referred to local referral hospitals or clinics.

8.1.2. Enrolment visit (at the start of week 1)

Women will be invited for the enrolment visit which will take place on the first day of their menstrual cycle. (ie. the first day of menstrual bleeding). A visit window of + 1 day will be allowed when the menstrual cycle starts on a Sunday or bank holiday. The visit can be rescheduled maximum once if the participant is not on her first day of menstrual bleeding (+ 1 day).

All test results from the baseline visit will be reviewed, and eligibility will be confirmed. Eligible women will be randomized either to an intermittent regimen (group A) or an extended (continuous) regimen (group B) of ring use.

There will be no re-screenings.

The following procedures will also be carried out:

- The general physical examination will be performed as at baseline.
- All women will be offered risk reduction counselling and condoms free of charge.
- A urine sample will be obtained for pregnancy testing.
- Study staff will teach women how to use the ring and study participants will be asked to insert the ring while still at the study clinic.

- A questionnaire on acceptability of the ring after the ring has been inserted.
- Participants will receive a diary card and will be explained how to complete a diary card regarding ring use and sexual behaviour as well as how to complete a self-rating adherence scale.
- Provide a follow-up visit date: 3 weeks after the enrolment date.

8.1.3. Follow up visits: Visits 2 to Visits 6

The follow up visits are planned to coincide with times of removal and insertion of the ring. Women in group A will visit the clinic at days of removal and insertion. These visits will coincide in group B as the old ring will be replaced by a new one at the same visit.
Participants in group B will be allowed to complete each follow-up visit up to three days prior/after their scheduled visit. The visit window for participants in group A (intermittent use) will depend of the kind of visit. Ring removal visits can be delayed for a maximum of 2 days, ring insertion visits can be performed a maximum of 2 days in advance. Every effort should be made to ensure participants adhere to this visit schedule.

<u>The following procedures are performed at each ring removal visit (both study arms):</u>

- Information on adverse events, social harms and concomitant medication will be collected.
- The pelvic and general physical examinations will be performed as at baseline. A total of 3 vaginal swabs are collected for:
  - One cotton swab for Wet Mount [*Trichomonas vaginalis* (TV); *Candida albicans*] and smears for BV diagnosis;
  - Two regular flocked swabs (Copan, Italy) for the characterisation of the vaginal microbiome.
- At visit 6 for Group A and visit 5 for Group B, after the final ring has been removed and all other vaginal specimens have been collected, a CVL sample will be collected.
- A urine sample is obtained, tested for pregnancy and an aliquot of fixated urine will be stored for FISH analysis.
- A completed diary card will be collected at each visit and a new diary card will be provided. Any problems with ring use will be addressed.
- The removed ring will be processed according to the instructions provided by the ITM and stored for biofilm analysis.
- Risk reduction counselling will be provided as well as condoms (free of charge).
- During the last ring removal visit (V6 for group A and V5 for group B) the following additional procedures will be offered:
  - a Visual Inspection with Acetic acid (VIA) to identify women at risk for cervical cancer;
  - An HIV test according to the national HIV algorithm. A maximum of 5 ml of blood will be collected by venepuncture in a serum tube. Pre- and post-test HIV counselling will be provided.

Additional questionnaires during ring removal visits:

1. The IAQ will be performed at every visit and will assess adherence and acceptability of the ring use but will be more comprehensive at visits 4 and 6 for group A and at visits 3 and 5 for group B. It will also assess factors that might influence the vaginal microbiome and vaginal inflammation.
2. Between weeks 7 and 9, 16-24 women will be enrolled in one of two FGD in each randomization group, for a total of four FGD.

3. At the last ring removal visit, and if funding permits, all study participants will be invited to provide their feedback on experiences with the new contraceptive method, on acceptability and adherence using ACASI.

<u>The following procedures are performed at the ring insertion visits for group A:</u>

- Information on adverse events, social harms and concomitant medication will be collected.
- Risk reduction counselling will be provided as well as condoms (free of charge).

8.1.4. Final Study Visit

At the last visit for both groups at week 14 ± 3 days, a sub-sample of women will be invited to participate in IDIs (3-5 women) and FGDs (8-12 women in each group) until data saturation is reached. Women participating in IDIs will be purposively selected among women who agreed to participate in IDIs and according to age/parity and experience with contraception in order to have a wide variation of key characteristics and experiences.
Women participating to FGDs will be purposively selected among women participating in the clinical trial and who have previously accepted to be included in the qualitative research, according to the same criteria as IDIs, i.e. age/parity and experience with contraception.

IDIs will also be conducted with male partners (n=5 in each randomization group). Women who participated to the after-trial FGD or IDIs will invite their partner to participate in IDIs. Those who agree to participate will be included in the end trial IDIs to reach a sample of 5 in each randomization group.

8.1.5. Unscheduled Visits

Participants may make interim contacts and unscheduled visits at their request, or as deemed necessary by the Principal Investigator or designee, at any time during the study. All unscheduled visits will be documented in the participant’s study file and on the study CRFs.

8.1.6. Missed Visits

Clinic staff will obtain contact information for each participant. In the event that a participant missed a scheduled appointment, study staff will immediately try to establish communication with the participant in accordance with the RU retention SOP. Mediums for contacting a participant should include only those approved by the participant beforehand, and should be implemented in such a way as to protect her confidentiality and privacy. All attempts to contact participants will be documented on the participant’s contact form which is kept in the Outreach Manager’s office until study completion. The need to complete all scheduled follow-up visits will be emphasized to all study participants at every visit. If the participant misses a scheduled visit but comes back outside the visit window for an unscheduled visit, she will receive additional counselling, will be asked to use dual protection and will receive a new appointment schedule.

8.1.7. Early discontinuation visit

When a participant is early discontinued from the study, every reasonable effort will be made to conduct all procedures of the last ring removal visit.

8.1.8. Schedule of assessments

	Group A			Group B		
		Ring in/out	Procedures		Ring in/out	Procedures
Pre-screening			HIV/STI Counselling<br>Informed Consent<br>Collect contact- and menses information			HIV/STi Counselling<br>Informed Consent<br>Collect contact- and menses information
IDI			IDI			IDI
Baseline (Week 0)			Current Medication/Medical History/Counselling<br>Vaginal/physical exam<br>Blood/vaginal (swabs + CVL)/ urine samples<br>IAQ			Current Medication/Medical History/Counselling<br>Vaginal/physical exam<br>Blood/vaginal (swabs + CVL)/ urine samples<br>IAQ
Enrolment (Week 1)	V1	Ring 1 in	Counselling<br>Randomization<br>Physical exam<br>Urine sample	V1 (±3d)	Ring 1 in	Counselling<br>Randomization<br>Physical exam<br>Urine sample
Week 4	V2 (+2d)	Ring 1 out	CM/AE/Counselling<br>Vaginal/physical exam<br>Vaginal (swabs)/urine samples<br>IAQ	V2 (±3d)	Ring 1 out/2 in	CM/AE/Counselling<br>Vaginal/physical exam<br>Vaginal (swabs)/urine samples<br>IAQ
Week 5	V3 (-2d)	Ring 2 in	CM/AE/counselling			
Week 7				V3 (±3d)	Ring 2 out/3 in	CM/AE/Counselling<br>Vaginal/physical exam<br>Vaginal (swabs)/urine samples<br>IAQ<br>FGD
Week 8	V4 (+2 d)	Ring 2 out	CM/AE /Counselling<br>Vaginal/physical exam<br>Vaginal (swabs)/urine samples<br>IAQ<br>FGD			
Week 9	V5 (-2d)	Ring 3 in	CM/AE/Counselling			
Week 10				V4 (±3d)	Ring 3 out/4 in	CM/AE/Counselling<br>Vaginal/physical exam<br>Vaginal (swabs)/urine samples<br>IAQ
Week 12	V6 (+2 d)	Ring 3 out	CM/AE /Counselling<br>Vaginal/physical exam<br>VIA<br>Blood/vaginal (swabs + CVL)/ urine samples<br>ACASI<br>IAQ			
Week 13				V5 (±3d)	Ring 4 out	CM/AE/Counselling<br>Vaginal/physical exam<br>VIA<br>Blood/vaginal (swabs + CVL)/ urine samples<br>ACASI<br>IAQ
Week 14	V7 (±3d)		FGD/IDI	V6 (±3d)		FGD/IDI

Green cells: study site visits for group A; Blue cells: study site visits for group B
CM/AE: Concomitant Medication/Adverse Events
IDI: In-Depth Interview
IAQ: Interviewer Administered Questionnaire
FGD: Focus Group Discussion
VIA: Visual Inspection with Acetic acid

8.2. Obtaining informed consent

All informed consent procedures (including assessment of understanding and literacy assessment) will be conducted by qualified staff members identified by the Principal Investigator and done in the language chosen by the participants. Participant Information Sheets and consent forms in the local language will be provided to the study participants or Legally Authorized Representatives and witnesses for their review. After the informed consent procedure, the participants and, in case they are of minor age (age below 21 in Rwanda), the parents or guardians will be asked to confirm their willingness to participate in the study by signing (or thumb-printing whenever they are illiterate) the consent form. The RU SOP for Informed Consent will be followed. Women who are between 18 and 21 but are legally married are no longer considered minors and do not require a guardian or parent to confirm their participation.
Separate IC forms will be given to all study participants who agreed to participate in FGDs and/or IDIs. Additionally, partners of study participants who agreed to participate in the after trial FGD and IDIs will also need to sign an informed consent form. The informed consent procedure will describe the purpose of the study, the procedures to be followed, the risks and benefits of participation, etc., according to the requirements described in the Helsinki Declaration, in Rwandan legislation and in the ICH GCP guidelines. If a participant (or parent or guardian) is unable to read or write, an independent witness will take part in the informed consent discussion and his/her signature will be obtained. Participants will be informed that participation in the study is completely voluntary and that they may withdraw from the study at any time without any negative consequences. If the woman is minor and unmarried, the consent must be given by a parent or legal guardian according to national law; however, in this case, the investigator is responsible to check that the participant is also freely willing to take part in the study. Therefore, minors will also sign the consent form, to document that they are freely giving their assent to be included in the study.

At the pre-screening and enrolment visits, the participants will be asked orally and in the IC form to consent for long term storage of samples (5 years after final study report) for future testing, with the exception of genetic testing. Future testing may include repeat and confirmatory testing to arrive at a proper diagnosis, testing that is already specified in this protocol but will be done on batched samples after all samples have been collected, or additional research related to the study objectives that is formulated by the study investigators in a substantial amendment and approved by the sponsor and the same ethics committees which approved the main protocol. If a participant refuses consent for long term storage, her specimens will be destroyed after the submission of the final study report.

8.3. Specific data collection procedures

Due to the multidisciplinary research objectives of the study, data is collected in different databases (refer to section 13. Data management). The data collection procedures for these different databases are explained in this section.

8.3.1. Quantitative data collection procedures

All participants will be interviewed using a structured questionnaire with questions including but not limited to demographics, sexual and contraceptive behaviour, reproductive and medical history, and factors that might influence the vaginal microbiome and inflammation, as well as adherence and acceptability of the ring. All the items which are relevant to the

clinical trial will then be transcribed on the eCRF (electronic-CRF) of the clinical trial. Results of the physical/genital examinations and laboratory results related to the primary and secondary clinical objectives that are available during data collection will be registered in the eCRFs. Laboratory results related to samples analysed in batches after all samples have been collected will be entered in separate databases according to the study SOPs at the institutions where the laboratory analyses will be carried out, and these databases will be merged with the main clinical trial database at the end of the study.

IAQs will be used *during the trial* by a trained interviewer to assess acceptability and adherence among all study participants. Questions will cover main attributes related to the direct user experiences as they will have emerged from the qualitative research (including ease of use, side effects, level of satisfaction and partner's experiences). The questionnaire to assess adherence and acceptability of the ring use will be more comprehensive at visit 4 and 6 for group A and at visit 3 and 5 for group B.

During the trial participants will be asked to complete a **diary card** regarding ring use and sexual behaviour as well as complete a self-rating adherence scale.

8.3.2. Qualitative data collection procedures

Among a selected sample of clinical trial participants the following interviews/questionnaires will take place and this information will be registered on the qualitative CRF.

IDIs will be conducted by trained interviewers *before* the start of the clinical trial, and will assess the following domains (in accordance with Woodsong et al)[9]:

1. Attributes relevant to the user experience (experiences with contraceptives, convenience, ease of use, perceived safety, beliefs about side-effects of contraceptives, relevance of contraceptive and HIV prevention safety);
2. Relationship attributes (partner support; couple communication and negotiation about contraceptives and negotiation);
3. Sexual encounter attributes (interference with sexual activity and pleasure);
4. Contextual factors (gendered norms, perceived community norms on contraceptives and sexuality and kinship and family structure);
5. Meaning of menstruation;
6. Risk and risk perceptions regarding unwanted pregnancy and HIV prevention.

Findings from these in-depth interviews will be used to construct the questionnaires that will be used at baseline and at the follow-up visits, and to adapt the theoretical framework, if needed.

ACASI will be used during *the last ring removal visit* (if funding permits) to get a complementary feedback on experiences, acceptability and adherence of the vaginal ring. Several key questions already asked during IAQ will be repeated, allowing data comparison/triangulation and assessing the validity of the data.

FGDs will be held *midway through* and *after* the trial with purposively selected groups of study participants. These FGDs will serve to get further insight on acceptability and user-experience findings. After the trial FGDs will also serve to clarify inconsistencies that have emerged from the clinical trial. In addition, the FGD will assess practical recommendations based on the users' experiences on how best to support future users with achieving high adherence and overcoming practical barriers with vaginal product use (such as insertion and

removal outside the clinic setting). The purpose of the additional FGDs with women who are not participants in the clinical trial (family planning users and women living with HIV) is to assess compatibility of vaginal ring use with certain aspects of Rwandan culture such as sexual behaviours, menses, fertility, and vaginal hygiene practices. We will get a more comprehensive view of these aspects of Rwandan culture when questions are not only asked in the context of a clinical trial setting and vaginal ring use but also in non-trial populations of potential future vaginal ring users.

Additional **IDIs** will be held after the trial to assess the methods' influence on sexuality, such as sexual pleasure, beliefs around sexuality, partner's influence. This concluding part of the qualitative research triangulated with the other research findings, will also contribute to examine the appropriateness of the theoretical framework used.

Among partners of study participants, **IDIs** will be conducted by trained interviewers with a selected number of partners of women having participated in the 'after trial FGD or IDIs' and will assess the following domains (in accordance with Woodsong et al):

1. Relationship attributes (partner support; couple communication and negotiation about contraceptives and negotiation);
2. Sexual encounter attributes (interference with sexual activity and pleasure);
3. Contextual factors (gendered norms, perceived community norms on contraceptives and HIV prevention and kinship and family structure).

Gender-adapted versions of the in-depth interview guides will be used.

8.4. Laboratory procedures

All treatable genital infections that are identified using the syndromic approach and any infections that were missed syndromically but identified later by laboratory testing will be treated according to RU SOPs that are based on national guidelines and available resources.

The following diagnostic tests will be performed in the laboratory at RU or other laboratories in Rwanda if at all possible, and at the STI reference laboratory of ITM if the capacity cannot be found in Rwanda, using validated test kits or methods, and procedures.

- Urine analysis for pregnancy using an urine rapid pregnancy test;
- HIV testing will follow RU SOPs, which are based on the Rwandan national guidelines;
- Wet Mount Microscopy;
- CT/NG molecular testing;
- HSV-2 and syphilis testing.

8.4.1. Research laboratory procedures at RU or in other laboratories in Rwanda

The following research tests will be performed at the RU laboratory:

1. A vaginal smear will be used for BV diagnosis and a microscopic description of the vaginal flora. The current gold standard for BV diagnosis in research settings is the Nugent score. This method relies on the semi-quantification of the bacterial morphotypes observed in a vaginal smear after Gram staining. The modified Ison & Hay grading is another system to describe the vaginal flora and provides more details on the *Lactobacillus* species compared to the Nugent score. The Nugent score will be used to diagnose BV and offer treatment to the women, whereas the modified Ison & Hay grading will be used for research purposes only.

2. Luminex cytokine testing will be used to identify women who have vaginal inflammation. All samples that are showing elevated cytokine levels, as well as an equal number of controls not showing elevated cytokine levels, will be tested further for the presence of proteins involved in inflammatory pathways.
3. An additional cervical swab will be taken at baseline for the rapid detection of CT using the BioChekSwab; a rapid self-contained, two step enzyme detection system. The test will be evaluated by comparing results obtained by this rapid test with the results obtained by the CT/NG molecular technique.

8.4.2. Research laboratory procedures at ITM

During the study, vaginal swabs, urine samples and the processed vaginal rings will be shipped to the STI reference laboratory at ITM for further testing:

1. Vaginal swabs will be tested with molecular amplification techniques for the characterization and quantification of the vaginal microbiome. Smears and an aliquot of the urine samples will be fixated and shipped to ITM to study the changes in phenotype of BV-related bacteria (from dispersed forms to adhesive forms on epithelial cells) to identify biofilm formation. Dispersed and adherent bacteria will be assessed in a multicolour analysis with a mix of differently stained group-specific and universal bacterial probes and visualized by confocal imaging.
2. The biofilms formed on the CVRs will be visualized using crystal violet staining and FISH, additional techniques may be applied if funding permits.

The shipment of the samples will be arranged by a regular courier by the laboratory administrator after proper preparation. Biological materials will be prepared and shipped in compliance with any applicable legislations and a Material Transfer Agreement (MTA).

8.4.3. Stored specimens

The left-overs of the urine, blood and vaginal/cervical specimens, collected during the clinical study will be stored at RU or at ITM as defined in the analytical plan. Aliquots of vaginal swabs, blood, and urine will remain at RU as back-ups. These specimens may be used for repeat and confirmatory analyses to arrive at a proper diagnosis and/or for future research testing (see above). These study samples will be destroyed after the final study report has been issued, except as indicated in "8.2 Obtaining Informed Consent".

9. STUDY INVESTIGATIONAL PRODUCT

9.1. Purchasing, preparation and administration

NuvaRing® is manufactured by N.V. Organon (a subsidiary of Merck & Co., Inc.,), Oss, the Netherlands. The CVRs will be bought by the sponsor via an authorized pharmaceutical supplier in Belgium. Shipment of the CVRs to RU will be performed according to good distribution practices and after the import authorization is obtained by Rwanda competent authority. The CVRs will be shipped under refrigerated conditions (2-8°C) and the temperature will be monitored during the shipment.
NuvaRing® contains 11.7 mg etonogestrel and 2.7 mg ethinylestradiol. The CVR releases etonogestrel and ethinylestradiol at an average amount of 0.120 mg and 0.015 mg, respectively per 24 hours, over a period of 3 weeks.
The CVR is approved since 2001 by most countries within the European Union, and in the United Stated by the FDA.

9.2. Subject compliance monitoring

Participants will be asked to insert and remove the CVR during study visits. The CVR can be removed before sexual intercourse but needs to be replaced as soon as possible to ensure hormonal contraception. Participants will be asked to complete questionnaires (IAQ during the trial and ACASI at the end of the trial) including adherence related questions.

9.3. Concomitant therapy

NuvaRing® should not be used when the following medications are taken:

- Other hormonal contraceptives
- Medicinal products that induce microsomal enzymes, which can result in increased clearance of sex hormones
- Use of antibiotics such as penicillins and tetracyclines.

9.4. Packaging

Both arms will receive the same product in their original commercial packaging, labeled in English. An additional study leaflet with information on how to use the CVR will be provided to all study participants in a language that is understandable to her.

9.5. Reception, storage, dispensing and return

The investigational product (NuvaRing®) will be stored in a refrigerator (2°C – 8°C) before dispensation. The temperature of the refrigerator will be daily monitored during the week and documented.

The CVR will be removed from the participants during a site visit. The CVRs will be placed in fixation solution and stored in the refrigerator. At the end of the study, all CVRs will be shipped at ambient temperature to the ITM.

10.SAFETY ASSESSMENT

10.1. Adverse events

Safety and tolerability of the treatments will be evaluated by recording Adverse Events (AEs) and grading laboratory and vital signs evaluations in the CRF.

10.1.1. Definition of an adverse event

An AE is any untoward medical occurrence in a subject or clinical investigation subject administered a pharmaceutical product and that does not necessarily have a causal relationship with this treatment. An AE can therefore be any unfavourable and unintended sign (including an abnormal laboratory finding), symptom, or disease temporally associated with the use of a medicinal product, whether or not related to the medicinal product.

10.1.2. Severity, relationship of event to study drug, and outcome

The severity of a clinical adverse event is to be scored according to the following scale:

1	Mild	Awareness of sign or symptom, but easily tolerated
2	Moderate	Discomfort enough to cause interference with usual activity

3	Severe	Incapacitating with inability to work or perform usual activity
4	Life-threatening	Patients at risk of death at the time of the event

The relationship of an adverse event to study drug is to be assessed according to the following definitions and can only be done by the study physician:

1 Definitely unrelated

It should be reserved for those events which cannot be even remotely related to study participation (e.g. injury caused by a third party).

2 Unlikely

There is no reasonable temporal association between the study drug and the suspected event and the event could have been produced by the subject's clinical state or other modes of therapy administered to the subject.

3 Possible

The suspected adverse event may or may not follow a reasonable temporal sequence from study drug administration but seems to be the type of reaction that cannot be dismissed as unlikely. The event could have been produced or mimicked by the subject's clinical state or by other modes of therapy concomitantly administered to the subject.

4 Likely

The suspected adverse event follows a reasonable temporal sequence from study drug administration, abates upon discontinuation of the drug, and cannot be reasonably explained by the known characteristics of the subject's clinical state.

5 Definitely related

It should be reserved for those events which have no uncertainty in their relationship to test drug administration: this means that a re-challenge was positive.

The outcome of each AE must be assessed at the same visit and according to the following classification:

- Completely recovered :	The patient has fully recovered with no observable residual effects
- Not yet completely recovered :	Improvement in the patient’s condition has occurred, but the patient still has some residual effects
- Deterioration :	The patient’s overall condition has worsened
- Permanent damage :	The AE has resulted in a permanent impairment
- Death :	The patient died due to the AE
- Ongoing :	The AE has not resolved and remains the same as at onset
- Unknown :	The outcome of the AE is not known because the patient did not return for follow-up (lost to follow-up)

Changes in the severity of an AE will be documented to allow an assessment of the duration of the event at each level of intensity. Adverse events characterized as intermittent require documentation of onset and duration of each episode.

The investigator is obliged to assess the relationship between investigational product and the occurrence of each AE/SAE. The investigator will use clinical judgement to determine the relationship. Alternative causes, such as natural history of the underlying diseases, concomitant therapy, other risk factors, and the temporal relationship of the event to the NuvaRing® will be considered and investigated.

10.1.3. Adverse Event Management

Participants will be asked to inform the study staff of any medical problems while they are taking part in the study. Participants will be provided with contact information and instructions to contact the study physician or designee to report any AEs they may experience. AEs will be collected until the final ring removal visit and may also be identified during laboratory testing, medical histories and physical examinations. Treatment for AEs possibly/probably/definitely related to study participation will be provided by the study clinic, to the extent possible, at no cost to the participant.

All clinical and laboratory toxicities will be managed according to standard medical guidelines.

All AEs will be followed up until resolution.

10.1.4. Pregnancy outcomes

Should a participant become pregnant during the study, she will be followed up by the study team until delivery, and the pregnancy outcome will be collected and documented. Any pregnancy outcomes that meet criteria for SAE reporting (e.g. congenital anomalies) occurring among participants will be reported as SAEs to the concerned bodies and included in the study report.

10.1.5. Serious Adverse Events (ICH E6, Section 1.50)

An SAE is any adverse event/experience occurring at any study drug dose that results in any of the following outcomes:

- Death;
- Life threatening (subject at immediate risk of death);
- Requires in-patient hospitalization or prolongation of existing hospitalization;
- Results in congenital anomaly/birth defect;
- Results in a persistent or significant disability or incapacity.

An important medical event that may not result in death, be life threatening, or require hospitalization may be considered an SAE when, based upon appropriate medical judgment, the event may jeopardize the subject and may require medical or surgical intervention to prevent one.

There may be situations when an SAE has occurred and the investigator has minimal information to include in the initial report to the Sponsor. However, it is very important that the investigator always makes an assessment of causality for every event prior to transmission of the report to the Sponsor according to the definitions in section 10.1.2. The investigator

may change his/her opinion of causality in light of follow-up information, amending the SAE-report form accordingly.

All SAE's whether or not deemed drug related or expected must be reported immediately or within 24 hours (one working day) using the SAE Notification Form by telefax or email to:

Clinical Trials Unit
Institute of Tropical Medicine
Nationalestraat 155
B-2000 Antwerp – Belgium
Fax: +32 3 247 66 47
Email: pharmacovigilance@itg.be

Line listings of all reported SAEs will be sent to the IRB of the ITM and the EC of UZA annually. All SAEs will be sent to the Rwanda National Ethics Committee case by case. RU will also submit annual line listings of all AEs to the Rwanda National Ethics Committee.

10.2. Data and Safety Monitoring Board

Due to the expected short recruitment period and the use of commercially available and approved CVRs, no formal Data Safety Monitoring Board (DSMB) will be set up. However, an independent data safety monitor (gynecologist) will be appointed who will receive, as they occur, and review all SAE reports with special attention to cardiovascular events (for example venous thrombosis) which may be associated with the use of hormonal contraception. In case of major safety concerns, this independent safety data monitor may request the sponsor to halt recruitment of the trial and/or to organize a formal DSMB with a complete overview of all available safety data.

11.ANALYSIS METHODS

11.1. Clinical Data

The statistical analysis of the primary objective and secondary objective 1 of the clinical trial will be performed by the study statistician at the CTU (ITM) according to a Statistical Analysis Plan which will be approved before database lock. The statistical analysis of secondary objectives 2 and 3 will be done by PhD student, at the ITM and at RU/UoL respectively, under the guidance of statisticians at the ITM and UoL. As these analyses will be research and data driven, no prior statistical analysis plan will be made but the statistical analysis methods will be fully described in the resulting reports and/or publications.

11.1.1. Study Objectives and Variables of Interest

The primary and secondary objectives of this clinical trial are to assess the impact on the vaginal microbiome of the use of a vaginal ring intermittently (3 weeks followed by 1 week off) or continuously (primary objective), to assess the general safety of the CVR (secondary objective 1), to assess vaginal biofilm formation and, to detect the presence or absence of a biofilm on the CVRs after intermittent or continuous use (secondary objective 2), and to determine the impact of intermittent or continuous use of the CVR on markers of inflammation and immune activation in the vagina (secondary objective 3).

The objectives will be reached by describing and comparing the variables of interest in women randomized to either intermittent vaginal ring use (group A) over a 12 week study period or continuous ring use (group B) over a 13 week study period.

The variables of interest are:

Primary: Changes from baseline (pre-ring use) in vaginal bacterial counts and in the absence/presence of BV-related bacteria as measured with quantitative real time PCR. Specifically, the flora species under study will include (but may not be limited to) the following species

a. Total *Lactobacillus* count
b. *Lactobacillus crispatus*
c. *L. iners*
d. *L. jensenii*
e. *L. gasseri*
f. *L. vaginalis*
g. *Atopobium vaginae*
h. *Gardnerella vaginalis*

Secondary:

1. Incidence of self-reported vaginal symptoms and clinician-observed signs Specifically:
    a. Incidence of self-reported urogenital symptoms: any ring-use emergent cases of irritation, burning, itching, discharge, bleeding, pain
    b. Incidence of genital signs on examination: any ring-use emergent cases of vaginal discharge, erythema, edema, ulcer, condyloma, petechia, ecchymosis, peeling

2. Incidence of BV as defined as a Nugent score >6

3. The numbers of women prematurely withdrawing from the study or discontinuing ring use

4. Incidence of adverse experiences during ring use and serious adverse experiences during or within 7 days after ring use

5. The numbers of incident STIs (HIV, HSV-2, Syphilis, Trichomoniasis, Chlamydia, Gonorrhea) and candidiasis

6. Nugent scores and Ison & Hay grading of the vaginal flora

7. Changes in phenotype of BV-related bacteria: specifically
    i. Presence of dispersed forms of bacteria
    ii. Presence of adhesive forms of bacteria on epithelial cells

8. Presence or absence of a biofilm on the CVRs after intermittent or continuous use
    b. Presence of biofilm visualized by crystal violet
    c. Visualization of adherent bacteria using FISH
    d. Identification of adherent bacteria (including but not limited to *Lactobacilli sp.*, *G. vaginalis, A. vaginae*)

9. Changes in inflammatory cytokines concentrations in CVLs as measured by Luminex technology. The cytokines analyzed include, but are not limited to IL-1, IL-6, IL-8, IL-10.

11.1.2. Statistical methods

11.1.2.1. Analysis populations

In general, analyses will be performed using an "as-randomized" all-patients-treated approach. All women randomized to either group and who insert the CVR at the enrollment visit will be included in the analysis under the group they are randomized to, even if they discontinue before visit 2 (week 4). For the analyses requiring vaginal or urine samples, all women with available data will be used, i.e. women without a sample prior to or during CVR use will be excluded.

11.1.2.2. Baseline characteristics

The number of women screened and enrolled or excluded will be summarized by reason for exclusion. For the women enrolled in the study, the number of women discontinued or lost-to follow-up will be tabulated by reason and visit of study discontinuation. This information will be summarized in a CONSORT flow diagram.

Patients in each treatment group will be described with respect to baseline characteristics. The description will be in terms of medians and interquartile ranges for continuous characteristics and using counts and percentages for categorical characteristics. The clinical importance of any imbalance will be noted but statistical tests of significance of baseline imbalance will not be undertaken.

11.1.2.3. Primary analysis

The primary analysis is based on the qPCR vaginal flora data. This data comprises different species and is recorded at baseline (week 0), and at 3 (group A) or 4 (group B) time points during CVR use.

The presence/absence of each species during CVR use will be analyzed using repeated measures logistic regression models, with random intercepts and random slopes over time for each woman and fixed effects for randomization group (intercept and slope).

For species which are present in the vaginal microbiome of the majority of women and remain present throughout the study, mixed-effects linear regression models will be fitted on the log-transformed bacterial counts. As in the logistic regression models, random intercepts and random slopes over time for each woman and fixed effects for randomization group (intercept and slope) will be included in the linear regression model.

In addition, overall counts of different species may be pooled (e.g., of all *Lactobacillus* species, or all *Lactobacillus* species excluding *L. iners*) and analyzed using mixed-effects linear regression. As an alternative approach, a principal component analysis (PCA) will be performed on the qPCR data and the PCA scores analyzed using mixed effects linear regression. It is expected, based on preliminary analyses of previous dataset, that the first principal component of the flora composition may indicate a gradient from a healthy to a less stable microflora. Changes in the scores for this principal component may consequently

indicate deleterious changes in the microbiome. Changes in the second and third principal component will also be explored if they appear to have a reasonable interpretation.

Effects over time will be described as odds-ratio's or changes in log-counts over each 4 week period together with 95% confidence intervals. Effects of the randomization group will be tested at the 5% level (two-sided).

11.1.2.4. Secondary analyses

The incidence of signs and symptoms, study/ring use withdrawals, adverse experiences, and STIs will be compared among randomization groups over the full study period using Fisher's exact test. Changes in Nugent scores and Ison & Hay grading of the vaginal flora will be graphically depicted and analyzed using repeated measures (logistic) regression models.

Analyses of endpoints related to secondary objectives 2 and 3 will be research and data driven. Presence/absence of dispersed/adherent form of BV-related bacteria and of biofilms will be analyzed using repeated measures logistic regression models.

Analyses of CVLs will focus on changes from baseline in cytokine concentrations. Care will be taken to mitigate the influence of truncation of the concentration data due to samples below or above the quantification limit of the assays used. Data reduction techniques as PCA may be used to summarize the multidimensional data in a limited number of variables. All samples that are showing elevated cytokine levels, as well as an equal number of controls not showing elevated cytokine levels, will be tested further for the presence of other proteins involved in inflammatory pathways. Control samples will be selected matched on major prognostic factors.

11.1.3. Sample size and power

The clinical study sample size calculation is based on the primary objective to assess the pre-post changes in the vaginal microbiome. We require 95% power to detect clinically important changes in bacterial counts. Preliminary analyses of our on-going biomarkers study showed a difference of (-)1 log in overall *Lactobacillus* sp. counts and of (+)3 log in *G. vaginalis* counts between participants with and without BV as assessed by Nugent score, and standard deviations of 1 and 3 for changes in overall *Lactobacillus* sp. counts and *G. vaginalis* counts, respectively. We have defined a change in bacterial count of 50% of the observed differences between women with and without BV as clinically important. Given this, we require 52 women in each study group. To correct for early withdrawals and women lost-to-follow up, we will randomize 120 women (60 in each study group) to ensure we have 104 women with primary endpoint data available.

11.2. Qualitative Data

All IDIs and FGDs will be audio-recorded, transcribed verbatim and translated into English. A debriefing session will be held after each IDI and FGD. Data will be uploaded into Nvivo 9.

For the primary objectives:

To analyze the IDI and FGD data we will use a deductive, content-analytical approach to assess if the components of the theoretical framework developed by Woodsong (2008)[9] are valid in the given setting. New elements that may emerge from the data will be integrated into the framework.

Selected statistical analysis will be conducted on the data of the questionnaires, the ACASI data (if available), the diary cards and a self-rating adherence scale. This quantitative analysis will be largely descriptive.

For the secondary objective:
To analyse the IDI and FGD we will use open coding methodology based on a framework developed from literature by the research team supplemented as themes emerge.

For the exploratory objectives:
To analyse the IDI and FGD we will use open coding methodology based on a framework developed by the research team from literature, including elements of Woodsong (2008) framework that might be relevant, by the research team supplemented as themes emerge.

All coding and analysis will be performed by at least two qualitative researchers to compare their coding and reach consensus on convergent issues.

In addition: Data from different sources such as the questionnaires, the ACASI data (if available), the diary cards, the data obtained from the IDI and FGD will be used to triangulate and assessing the validity of the data.

12.MONITORING AND QUALITY ASSURANCE

This study will be monitored in accordance with regulations applicable to clinical trials, including ICH-GCP and GCLP (Good Clinical Laboratory Practices) requirements, and sponsor-specific SOPs. The social science component research will adhere to the guidelines established by the American Anthropological Association. The objectives and specific tasks of the monitor are described in the ICH Guidelines E6. The details of the monitoring, which will be conducted and supervised by the Clinical Trial Unit (CTU) of the ITM, will be carried out according to the SOPs of the CTU of ITM.
The responsible monitor (or designee) will contact the site and visit the investigator regularly and will be allowed to inspect the various records of the study (CRFs and other pertinent data) provided that participant confidentiality is maintained in compliance with international and local regulations. It will be the monitor's responsibility to inspect the study facilities, the source documents and the CRFs at regular intervals throughout the study, to verify the adherence to the protocol and the completeness, consistency and accuracy of the data being entered on them, while it will be the responsibility of the PI to ensure that the monitor has full and timely access to all the study documents and facilities. The monitor should have access to laboratory test reports and other participant records needed to verify the entries on the CRF. The investigator (or his/her deputy) agrees to cooperate with the monitor to ensure that any problems detected in the course of these monitoring visits are resolved. Furthermore, the PI will ensure full and timely access to all study source and essential documents in case of a sponsor-initiated audit.

12.1. Laboratory quality control and quality assurance

A quality system was already put in place at RU by the ITM in previous collaborative projects and ITM will ensure that all laboratory activities including specimen transport, processing, testing, result reporting and storage will be conducted in accordance with the

clinical trial quality requirements. The GCLP guidelines will be followed and the laboratory will perform testing according to the SOPs which are documented in the analytical plan.
The on-site laboratory of RU will receive a hands-on training by the ITM a few days before and at the start of the study to ensure the readiness for study initiation. Thereafter, bi-yearly supervision visits to the laboratory will be organized. An external quality scheme will be set-up by the ITM for every diagnostic laboratory assay performed in Rwanda.

13.DATA MANAGEMENT

Due to the integration of quantitative and qualitative methods in this study 4 different types of databases will be set up.

- A clinical trial database will be programmed and validated prior to study start. If modifications are needed after the start of the study (e.g., in case of protocol amendment), testing and validation will be repeated.

- A quantitative behavioural/adherence/acceptability database that will also be programmed prior to study start. This database will be tested prior to use but not be fully validated according to a clinical trial validation protocol. Revisions to this database after study start may be necessary when the initial qualitative research shows that our questionnaires are not optimal.

- Separate laboratory databases will be developed for the laboratory tests that are done in batches after all samples have been collected. Data in these databases will be entered and double-checked manually by qualified laboratory professionals according to the study SOP. The data may be combined with variables from the above two databases for analysis.

- The qualitative behavioural/adherence/acceptability data (IDI/FGD transcripts and coding).

13.1. Data Management - Clinical Trial

Data Management of the clinical trial will be performed by the data manager at the CTU (ITM) and in collaboration with the study staff assigned for data management at the site (country coordinating investigator, PI, data entry clerk). As mentioned in 8.3.1., separate laboratory databases will be developed for laboratory objectives involving batched samples. These databases will be merged with the main study database at the end of the study.

13.1.1. Confidentiality and security of trial participant data (all databases)

Private information on trial participants will be handled confidential (see also section 14.4.). Information such as the participant name or any other data which could lead to the identity of the participant will not be included on the trial source document template, nor on the eCRF, nor on any other paper documents or electronic files used for data management.
Name and contact data for each participant will be kept separate and limited to the clinical staff at the site only.
All paper documents and electronic files needed for data management will be restricted to authorized study staff, both at the CTU and at the site. The study computers and eCRFs are only accessible via a LogIn with personal username and password. A list of authorized users of the eCRFs (and database) will be kept at the CTU and updated during the study.

13.1.2. Data Collection

For each trial participant, a file is provided at the site to store all original or source data for that person. These data will be registered in a paper source document template, especially designed for this trial and facilitating the organization, reconstruction and evaluation of the trial. Only data defined by the study protocol will be collected.
It is the PI or other assigned study staff who will fill in the source document templates and who will check for completeness or any irregularities before handing it over for data entry. If data are not available or missing then this will be explained in the source document template.

13.1.3. Data entry

Data registered on the source document template will be recorded via eCRFs into the clinical trial database, using OpenClinica. OpenClinica is the world's leading open source clinical trials software for electronic data capture (EDC) and clinical data management (CDM). OpenClinica supports ICH-GCP quality standards and guidelines and requirements such as 21 CFR part 11 (e.g. electronic signature, electronic audit trail). The software features amongst others an integrated CRF tracking and query management system and capabilities for importing and exporting data in various forms. Another major asset of OpenClinica is that the experience in the use of an open source data management system may be a valuable addition to capacity of the local site.

The eCRFs and database will be designed by the CTU data manager and mirroring the source document template. So called edit checks, programmed onto the eCRF will validate data at the point of data entry. Extensive system validation or testing of the eCRF will be done, in particular on the edit checks. It is only after approval of the testing and the Validation Report that the actual data entry may start.

Data will be entered at the site online. Trial participants will be identified by a study specific participant code and initials. All data entry and subsequent data handling will be done by trained study staff.

Laboratory databases for laboratory objectives involving batched samples will be completed following laboratory specific SOPs.

13.1.4. Data quality and checking

Ensuring data quality and data integrity is an essential task throughout the trial, with the CTU data manager in a coordinating role. Good practices during the design of the source document template and eCRF, such as use of coding, check boxes, question input masks and a drop down menu will enhance the data quality. Aside the automatic edit checks, also manual checks will be performed to identify out of range data, missing data and inconsistent data.

A system will be put in place to ensure timely data entry, data checking, resolving of queries and lastly database closure. Its details will be mentioned in the Data Management Plan. The CTU data manager and the concerned study staff will regularly hold conference calls on Data Management throughout the study.

13.1.5. Records keeping and Archiving (see also section 16)

Electronic documents for purpose of data management, including txt and pdf formats, Excel files, Access databases, mdb and ldf data files, eCRF generated files (especially study designs), programmes and emails are stored and organized on the ITM server in a study specific folder, in particular in a specific data management subfolder. Paper documents for purpose of data management, such as mail prints, data listings, data management documentation (see below) will be organized at the CTU in specific study binders. This principle will also be applied at the site.

Specific details on the minimum retention time for paper documents and electronic files, for CTU and site, can be found in section 16.

13.1.6. Data Management documentation

During the Pre-study phase, study phase and Post-study phase various documentation for the purpose of data management has to be produced. Essential documentation includes the Data Management Plan, Source Document Template, Data Entry Guidelines and System Validation Report. The CTU data manager will be responsible for producing and updating (if applicable) these documents.

14.ETHICAL ISSUES

14.1. Regulatory authorities and ethical review

The protocol and all study documents such as IC forms will be submitted for formal review and approval to the Institutional Review Board (IRB) of the ITM, the EC of the University Hospital of Antwerp (EC UZA) and the Rwandan National Ethics Committee (RNEC), the NHRC (National Health Research Committee) and to the Rwandan Ministry of Health (MoH). Relevant documentations will be submitted to the Rwandan MoH in order to obtain an approval for importing the vaginal rings. No study specific subject related activities will be performed before formal approval from all these bodies is obtained.

Copies of the national approvals and of the relevant correspondence will be transmitted from the investigator to ITM before starting recruitment. The ITM will transmit to the investigator the copies of approvals of the ITM IRB and of the EC UZA. Amendments to the protocol, including those related to additional analyses to be run with stored samples, must be approved and finalized by the Sponsor in agreement with the SC, and can only be implemented after formal approval from all concerned ECs and Competent Authorities (CA), as appropriate, unless immediate implementation is needed for subject safety reasons.
Yearly re-approval from the NREC must be obtained after an annual report will be submitted to the same institutions.

The study will be carried out according to the principles stated in the Declaration of Helsinki as amended in Seoul in 2008 and any further updates, all applicable national and international regulations and according to the most recent ICH and WHO GCP guidelines.

14.2. Protocol amendments

Once the final clinical study protocol has been issued and signed by the authorized signatories, it cannot be informally altered. Protocol amendments have the same legal status and must pass through the appropriate steps before being implemented. Any substantial

change must be approved by all the bodies and EC's that have approved the initial protocol prior to being implemented, unless it is due to participant's safety concerns, in which case the immediate implementation can be necessary for the sake of subject's protection. In case modifications to the protocol or amendment are requested by any local EC/CA during the review process, these must be discussed and agreed upon with the Sponsor prior to any resubmission incorporating those changes.

14.3. Informed Consent

No participant may be admitted into this study until the Site Investigator or designee has obtained his/her informed consent. The procedures how to obtain informed consent are described in section 8.2: Obtaining informed consent.

14.4. Confidentiality

All study documents are provided by the Sponsor to the Investigators and his/her appointed staff in confidence. None of this material may be disclosed to any party not directly involved with the study, without written permission from the Sponsor.

Each participant will be assigned a unique personal identification number, which will be used on all study forms. This identification number will be linked to the person's name and contact information in a central "subject identification list". The identification list and documents containing the names and/or signatures of participants (such as consent forms) will be kept separately from all other study documents containing participant data, that will only be identified by the participant identification numbers. All study documents will be stored in lockable rooms or cabinets with access limited to study staff.
All information obtained from the participants will be kept private to the extent allowed by the law. The findings of this study will be reported in summary form. Names of the participants will not appear on any reports or publications resulting from this study. The findings from this study may be published in a medical/scientific journal. The study participants will not be identified by name. After the study is completed, participants will be informed of the study results by the Principal Investigator.

All hard copy data from the different qualitative data collected such as notes, audio recordings, transcripts, etc. will be kept in a locked filing cabinet or equal secure location. The documents will be kept in one large envelope or file folder per event (FGD, IDI, etc...) with an archival cover sheet that lists all items included in the folder/envelope. No potential identifying information such as names or addresses (full or partial) will be written on any of the documents or envelopes, instead participant and staff ID's as well as archival codes will be used.

14.5. Risks and benefits

Study participants may feel discomfort and pain during pelvic exams or when undergoing phlebotomy. They also may feel dizzy or faint, and/or develop a bruise, swelling, or infection where the needle is inserted.
Participants may become embarrassed, worried or anxious during pelvic or physical exams, or when discussing their health, sexual behaviours, or as a result of being tested for HIV infection and counselled. They may also become worried or anxious while waiting for results

of their tests for HIV or other infections. They may experience anger or distress when they learn that they are HIV positive or that they have another STI. Counselling will be provided by counsellors who have been trained in specific issues related to HIV infection, including stigma, blame, methods to avoid transmission, and available support services.
Frequently reported undesirable effects of the NuvaRing® are headaches and vaginal infections but this only occurred in 5-6% of the women.

Although RU will make every effort to protect participant privacy and confidentiality, it is possible that participants involvement in the study could become known to others, and that social harms, including domestic violence, may result. RU has anticipated the range of social harms that might occur to participants (emotional, physical, financial), and has on site counselling as well as referral resources to meet the need that might arise. All social harms are documented and followed-up.

Participation in this study will result in free CVRs for minimum three months, maximum four months, depending in which group the participant is randomized. A few genital symptoms including vaginal wetness are associated with ring use [8]. It is also possible that the sexual partners can feel the ring during sexual intercourse. A study assessing the influence of vaginally and orally administered hormonal contraceptives on sexuality, found that both methods improved sexual functioning in women, compared to women not using any hormonal method [23].
At the end of the treatment period, every effort will be made to ensure that study participants will gain access to a reliable contraceptive method after they have completed study participation.

Other benefits are:
Participants will receive individual HIV and STI risk reduction counselling and will receive free HIV, STI and pregnancy testing, a pelvic and physical examination. They will also receive free medical treatments if they are available at the research centre and condoms. If the medical treatment is not available at the research centre, they will be referred to local public health facilities for treatment and care.

14.6. Compensation for Participation

The participants will be reimbursed for their travel to the study site at the end of each study visit that was scheduled by the RU study team. The specific amounts of reimbursement will be specified in the IC forms in the local currency and will be set in accordance with local guidelines. Reimbursement will also be provided for participation in any FGD or IDI. No financial compensation will be given to the participants for unscheduled visits that are judged not study-related by the RU study staff.

14.7. Insurance

The sponsor will obtain a no-fault liability insurance covering the entire conduct of the study. This insurance will cover the trial subjects and involved study staff for any damage or injury which results from any study-related activities or procedures. The insurance will also be mentioned during the informed consent discussion.

15. STEERING COMMITTEE AND TRIAL MANAGEMENT GROUP

A Steering Committee (SC) will be set up to manage the project and to supervise the clinical trial. The SC will consist of the protocol team roster, one representative of each partner institution, and a representative of the ITM Clinical Trials Unit (CTU). Other members of the project may join the meeting as external contributors.
The SC will have a planned teleconferencing meeting at the very start of the project and will have three monthly teleconferences to discuss the progress in each work package. The leaders of the different work packages will report to the SC and back to their teams and all decisions in the project regarding activities and resource allocation will be taken in the SC.

A Trial Management Group (TMG) will be set up according to the ITM procedures and it will be in charge of the day-to-day management of the clinical trial. It will include relevant members of the study team in Rwanda and in Europe.

16. ARCHIVING

The Investigator and the Sponsor must maintain adequate and accurate records to enable the conduct of the study to be fully documented and the study data to be subsequently verified.
At each site, these documents should be classified into two different separate categories: 1) Investigator's Trial File (IF), and 2) subject source documents. The Investigator's Trial File will contain the protocol/amendments, CRFs, Independent EC/IRB and governmental approval with correspondence, sample informed consent, staff curriculum vitae and other appropriate documents/correspondence as described in ICH GCP. The details are described in the CTU ITM SOP on Investigator File. Subject source documents may include physician and nurse's notes, original laboratory reports, signed IC forms etc.

All paper records will be stored securely in lockable filing cabinets at the clinical sites and will be archived for a minimum of 3 years from publication of the primary study results and in any case in accordance with current local regulations. The Sponsor will inform when records and specimens may be destroyed, and should be informed prior to destruction of the files and/or specimens. After completion of the study, the IF will remain available for internal audits and/or inspections of regulatory authorities for a period of twenty years, unless differently requested by national authorities.

The sponsor will maintain a Sponsor Trial Master File containing regulatory study documents and other appropriate documents, as described in ICH-GCP, during the course of the study. The details are described in the CTU ITM SOP on Trial Master File. At the conclusion of the study, the Trial Master File and copies of (e)CRFs will be archived for 20 years, in accordance with the Belgian legislation requirements and with the quality procedures of ITM.

17. REFERENCES

1. Malcolm RK, Woolfson AD, Toner CF, Morrow RJ, McCullagh SD: Long-term, controlled release of the HIV microbicide TMC120 from silicone elastomer vaginal rings. *J Antimicrob Chemother* 2005, 56:954-956.
2. Malcolm RK, Edwards KL, Kiser P, Romano J, Smith TJ: Advances in microbicide vaginal rings. *Antiviral Res* 2010, 88 Suppl 1:S30-S39.
3. Roumen FJ. Review of the combined contraceptive vaginal ring, NuvaRing. Ther Clin Risk Manag 2008; 4(2):441-451.
4. Barreiros FA, Guazzelli CA, Barbosa R, de AF, de Araujo FF. Extended regimens of the contraceptive vaginal ring: evaluation of clinical aspects. Contraception 2010; 81(3):223-225.
5. Brache V, Faundes A. Contraceptive vaginal rings: a review. Contraception 2010; 82(5):418-427.
6. Lopez L, Grimes D, Gallo M, Schulz K. Skin patch and vaginal ring versus combined oral contraceptives for contraception. Cochrane Database of Systematic Reviews 2010, Issue 3 Art No : CD003552 DOI: 10 1002/14651858 CD003552 pub3 2010.
7. Merkatz R. Nestorone/Ethinyl Estradiol Contraceptive Vaginal Ring - New User Controlled, Long Acting Contraceptive. International Conference on Family Planning: Research and Best Practices, Uganda, 15-18 November 2009.
8. van der straten A, Montgomery ET, Nel A, Cheng H, Wenger L, Young K, Romano J, Malherbe M, Woodsong C: High Acceptability of a Vaginal Ring as a Microbicide Delivery Method for HIV Prevention in African Women. AIDS Behav (2012) 16:1775-1786
9. Woodsong C S-RAAP. A comprehensive & flexible conceptual framework for investigating acceptability in microbicide clinical trials. Microbicides Conference 2008, (Abstract #TC-131).
10. Sha BE, Zariffard MR, Wang QJ, Chen HY, Bremer J, Cohen MH et al. Female genital-tract HIV load correlates inversely with Lactobacillus species but positively with bacterial vaginosis and Mycoplasma hominis. J Infect Dis 2005; 191(1):25-32.
11. van de Wijgert JH, Morrison CS, Brown J, Kwok C, Van Der PB, Chipato T et al. Disentangling contributions of reproductive tract infections to HIV acquisition in African Women. Sex Transm Dis 2009; 36(6):357-364.
12. Larsson PG, Brandsborg E, Forsum U, Pendharkar S, Krogh-Andersen K, Nasic S et al. Extended antimicrobial treatment of bacterial vaginosis combined with human lactobacilli to find the best treatment and minimize the risk of relapses. BMC Infect Dis 2011; 11(1):223.
13. Swidsinski A, Dorffel Y, Loening-Baucke V, Schilling J, Mendling W. Response of Gardnerella vaginalis biofilm to 5 days of moxifloxacin treatment. FEMS Immunol Med Microbiol 2011; 61(1):41-46.
14. Menard JP, Fenollar F, Henry M, Bretelle F, Raoult D. Molecular quantification of Gardnerella vaginalis and Atopobium vaginae loads to predict bacterial vaginosis. Clin Infect Dis 2008; 47(1):33-43.
15. Ravel J, Gajer P, Abdo Z, Schneider GM, Koenig SS, McCulle SL et al. Vaginal microbiome of reproductive-age women. Proc Natl Acad Sci U S A 2011; 108 Suppl 1:4680-4687.
16. Donlan RM. Biofilms and device-associated infections. Emerg Infect Dis 2001; 7(2):277-281.
17. Gunawardana M, Moss JA, Smith TJ, Kennedy S, Kopin E, Nguyen C et al. Microbial biofilms on the surface of intravaginal rings worn in non-human primates. J Med Microbiol 2011; 60(Pt 6):828-837.
18. http://www.ipmglobal.org/our-work/microbicide-research-development/steps-concept-product/developing-combination-microbicide-p.; 2012.
19. Dhont N, Ndayisaba GF, Peltier CA, Nzabonimpa A, Temmerman M, van de Wijgert J: Improved access increases postpartum uptake of contraceptive implants among HIV-positive women in Rwanda. *Eur J Contracept Reprod Health Care* 2009, 14:420-425.
20. Elul B, Delvaux T, Munyana E, Lahuerta M, Horowitz D, Ndagije F, Roberfroid D, Mugisha V, Nash D, Asiimwe A: Pregnancy desires, and contraceptive knowledge and use among prevention of mother-to-child transmission clients in Rwanda. *AIDS* 2009, 23 Suppl 1:S19-S26.
21. Leslie JA, Munyambanza E, Adamchak SE, Janowitz B, Grey TW, Kirota K: Without strong integration of family planning into PMTCT services in Rwanda, clients remain with a high unmet need for effective family planning. *Afr J Reprod Health* 2010, 14:144-146.
22. Morrison CS, Chen PL, Kwok C, Richardson BA, Chipato T, Mugerwa R, Byamugisha J, Padian N, Celentano DD, Salata RA: Hormonal contraception and HIV acquisition: reanalysis using marginal structural modeling. *AIDS* 2010, 24:1778-1781.
23. Guida M, Di Spiezio SA, Bramante S, Sparice S, Acunzo G, Tommaselli GA et al. Effects of two types of hormonal contraception--oral versus intravaginal--on the sexual life of women and their partners. Hum Reprod 2005; 20(4):1100-1106.

18. ANNEXES

Annex 1: Contact details of all collaborators

The following persons are currently involved in the study. In case of changes, an updated contact list will be sent to the ECs yearly.

Affiliation	Name	Function	Contact details
RU<br>KN 50th 716 – Kiyovu – Kigali – Rwanda	Dr. Stephen Agaba	Study Physician and principal investigator	Email:agabakarema@yahoo.co.uk<br>Tel: +250 788 35 78 66
	Ms. Evelyne Kestelyn	Country Coordinating Investigator - pre-doctoral social scientist	Email: e.kestelyn@aighd.org<br>Tel: +250 788 30 07 69
	Dr. Jean-Claude Ndagijimana	Study-physician and investigator	Email: ndagiclau@yahoo.fr<br>Tel: +250 788 42 26 65
	Mr. Lambert Mwambarangwe	Lab QC/QA Manager	Email: mwambara2@yahoo.fr<br>Tel: +250 728 41 49 60
	Ms. Mireille Uwineza	Study Coordinator	Email: uwinezamireille@yahoo.fr<br>Tel: +250 788 59 31 58
ITM<br>Nationalestraat 155 – 2000 Antwerp – Belgium	Dr Tania Crucitti	Coordinating investigator	Email: tcrucitti@itg.be<br>Tel: +32 3 247 65 52
	Dr. Vicky Jespers	Clinical Investigator	Email: vjespers@itg.be<br>Tel: +32 3 247 62 96
	Dr. Thérèse Delvaux	Leader Social Science Package	Email: tdelvaux@itg.be<br>Tel: +32 3 247 62 95
	Mr. Joris Menten	Statistics Coordinator	Email: jmenten@itg.be<br>Tel: +32 3 247 66 17
	Ms. Irith De Baetselier	Study Management Coordinator	Email: idebaetselier@itg.be<br>Tel: +32 3 247 65 44
	Ms. Raffaella Ravinetto	Head of the Clinical Trials Unit	Email: rravinetto@itg.be<br>Tel: +32 3 247 66 25
	Ms. Céline Schurmans	Monitoring Coordinator	Email: cschurmans@itg.be<br>Tel: +32 3 247 07 78
	Mr. Harry van Loen	Data Management Coordinator	Email: hvanloen@itg.be<br>Tel: +32 3 247 66 16
	Ms. Liselotte Hardy	Pre-doctoral scientist	Email: lhardy@itg.be<br>Tel: +32 3 247 65 49
	Ms. Danielle Van Melle	Clinical Research Assistant	Email: dvmelle@itg.be<br>Tel: +32 3 247 66 34
UoL<br>Ronal Ross Building, 8 West Derby Street, Liverpool, UK	Dr. Janneke van de Wijgert	Collaborator/Co-coordinating Investigator	Email: j.vandewijgert@liverpool.ac.uk<br>Tel: +44 755 719 51 08
	Dr. Jennifer Ilo Van Nuil	Post-doctoral social scientist	Email: Jennifer.Van-Nuil@liverpool.ac.uk<br>Tel: +250 786 41 91 85

Annex 2: World Medical Association Declaration of Helsinki

Ethical Principles for Medical Research Involving Human Subjects
Adopted by the 18th WMA General Assembly, Helsinki, Finland, June 1964, and amended by the:
29th WMA General Assembly, Tokyo, Japan, October 1975
35th WMA General Assembly, Venice, Italy, October 1983
41st WMA General Assembly, Hong Kong, September 1989
48th WMA General Assembly, Somerset West, Republic of South Africa, October 1996
52nd WMA General Assembly, Edinburgh, Scotland, October 2000
53th WMA General Assembly, Washington 2002 (Note of Clarification on paragraph 29 added)
55th WMA General Assembly, Tokyo 2004 (Note of Clarification on Paragraph 30 added)
59th WMA General Assembly, Seoul, October 2008

A. INTRODUCTION

1. The World Medical Association (WMA) has developed the Declaration of Helsinki as statement of ethical principles for medical research involving human subjects**,** including research on identifiable human material and data.

   The Declaration is intended to be read as a whole and each of its constituent paragraphs should not be applied without consideration of all other relevant paragraphs.

2. Although the Declaration is addressed primarily to physicians, the WMA encourages other participants in medical research involving human subjects to adopt these principles.

3. It is the duty of the physician to promote and safeguard the health of patients, including those who are involved in medical research. The physician's knowledge and conscience are dedicated to the fulfillment of this duty.

4. The Declaration of Geneva of the WMA binds the physician with the words, “The health of my patient will be my first consideration,” and the International Code of Medical Ethics declares that, “A physician shall act in the patient's best interest when providing medical care.”

5. Medical progress is based on research that ultimately must include studies involving human subjects. Populations that are underrepresented in medical research should be provided appropriate access to participation in research.

6. In medical research involving human subjects, the well-being of the individual research subject must take precedence over all other interests.

7. The primary purpose of medical research involving human subjects is to understand the causes, development and effects of diseases and improve preventive, diagnostic and therapeutic interventions (methods, procedures and treatments). Even the best current interventions must be evaluated continually through research for their safety, effectiveness, efficiency, accessibility and quality.

8. In medical practice and in medical research, most interventions involve risks and burdens.

9. Medical research is subject to ethical standards that promote respect for all human subjects and protect their health and rights. Some research populations are particularly vulnerable and need special protection. These include those who cannot give or refuse consent for themselves and those who may be vulnerable to coercion or undue influence.

10. Physicians should consider the ethical, legal and regulatory norms and standards for research involving human subjects in their own countries as well as applicable international norms and standards. No national or international ethical, legal or regulatory requirement should reduce or eliminate any of the protections for research subjects set forth in this Declaration.

B. PRINCIPLES FOR ALL MEDICAL RESEARCH

11. It is the duty of physicians who participate in medical research to protect the life, health, dignity, integrity, right to self-determination, privacy, and confidentiality of personal information of research subjects.
12. Medical research involving human subjects must conform to generally accepted scientific principles, be based on a thorough knowledge of the scientific literature, other relevant sources of information, and adequate laboratory and, as appropriate, animal experimentation. The welfare of animals used for research must be respected.
13. Appropriate caution must be exercised in the conduct of medical research that may harm the environment.
14. The design and performance of each research study involving human subjects must be clearly described in a research protocol. The protocol should contain a statement of the ethical considerations involved and should indicate how the principles in this Declaration have been addressed. The protocol should include information regarding funding, sponsors, institutional affiliations, other potential conflicts of interest, incentives for subjects and provisions for treating and/or compensating subjects who are harmed as a consequence of participation in the research study. The protocol should describe arrangements for post-study access by study subjects to interventions identified as beneficial in the study or access to other appropriate care or benefits.
15. The research protocol must be submitted for consideration, comment, guidance and approval to a research ethics committee before the study begins. This committee must be independent of the researcher, the sponsor and any other undue influence. It must take into consideration the laws and regulations of the country or countries in which the research is to be performed as well as applicable international norms and standards but these must not be allowed to reduce or eliminate any of the protections for research subjects set forth in this Declaration. The committee must have the right to monitor ongoing studies. The researcher must provide monitoring information to the committee, especially information about any serious adverse events. No change to the protocol may be made without consideration and approval by the committee.
16. Medical research involving human subjects must be conducted only by individuals with the appropriate scientific training and qualifications. Research on patients or healthy volunteers requires the supervision of a competent and appropriately qualified physician or other health care professional. The responsibility for the protection of research subjects must always rest with the physician or other health care professional and never the research subjects, even though they have given consent.
17. Medical research involving a disadvantaged or vulnerable population or community is only justified if the research is responsive to the health needs and priorities of this population or community and if there is a reasonable likelihood that this population or community stands to benefit from the results of the research.
18. Every medical research study involving human subjects must be preceded by careful assessment of predictable risks and burdens to the individuals and communities involved in the research in comparison with foreseeable benefits to them and to other individuals or communities affected by the condition under investigation.
19. Every clinical trial must be registered in a publicly accessible database before recruitment of the first subject.
20. Physicians may not participate in a research study involving human subjects unless they are confident that the risks involved have been adequately assessed and can be satisfactorily managed. Physicians must immediately stop a study when the risks are found to outweigh the potential benefits or when there is conclusive proof of positive and beneficial results.

21. Medical research involving human subjects may only be conducted if the importance of the objective outweighs the inherent risks and burdens to the research subjects.
22. Participation by competent individuals as subjects in medical research must be voluntary. Although it may be appropriate to consult family members or community leaders, no competent individual may be enrolled in a research study unless he or she freely agrees.
23. Every precaution must be taken to protect the privacy of research subjects and the confidentiality of their personal information and to minimize the impact of the study on their physical, mental and social integrity.
24. In medical research involving competent human subjects, each potential subject must be adequately informed of the aims, methods, sources of funding, any possible conflicts of interest, institutional affiliations of the researcher, the anticipated benefits and potential risks of the study and the discomfort it may entail, and any other relevant aspects of the study. The potential subject must be informed of the right to refuse to participate in the study or to withdraw consent to participate at any time without reprisal. Special attention should be given to the specific information needs of individual potential subjects as well as to the methods used to deliver the information. After ensuring that the potential subject has understood the information, the physician or another appropriately qualified individual must then seek the potential subject's freely-given informed consent, preferably in writing. If the consent cannot be expressed in writing, the non-written consent must be formally documented and witnessed.
25. For medical research using identifiable human material or data, physicians must normally seek consent for the collection, analysis, storage and/or reuse. There may be situations where consent would be impossible or impractical to obtain for such research or would pose a threat to the validity of the research. In such situations the research may be done only after consideration and approval of a research ethics committee.
26. When seeking informed consent for participation in a research study the physician should be particularly cautious if the potential subject is in a dependent relationship with the physician or may consent under duress. In such situations the informed consent should be sought by an appropriately qualified individual who is completely independent of this relationship.
27. For a potential research subject who is incompetent, the physician must seek informed consent from the legally authorized representative. These individuals must not be included in a research study that has no likelihood of benefit for them unless it is intended to promote the health of the population represented by the potential subject, the research cannot instead be performed with competent persons, and the research entails only minimal risk and minimal burden.
28. When a potential research subject who is deemed incompetent is able to give assent to decisions about participation in research, the physician must seek that assent in addition to the consent of the legally authorized representative. The potential subject's dissent should be respected.
29. Research involving subjects who are physically or mentally incapable of giving consent, for example, unconscious patients, may be done only if the physical or mental condition that prevents giving informed consent is a necessary characteristic of the research population. In such circumstances the physician should seek informed consent from the legally authorized representative. If no such representative is available and if the research cannot be delayed, the study may proceed without informed consent provided that the specific reasons for involving subjects with a condition that renders them unable to give informed consent have been stated in the research protocol and the study has been approved by a research ethics committee. Consent to remain in the research should be obtained as soon as possible from the subject or a legally authorized representative.
30. Authors, editors and publishers all have ethical obligations with regard to the publication of the results of research. Authors have a duty to make publicly available the results of their

research on human subjects and are accountable for the completeness and accuracy of their reports. They should adhere to accepted guidelines for ethical reporting. Negative and inconclusive as well as positive results should be published or otherwise made publicly available. Sources of funding, institutional affiliations and conflicts of interest should be declared in the publication. Reports of research not in accordance with the principles of this Declaration should not be accepted for publication.

C. ADDITIONAL PRINCIPLES FOR MEDICAL RESEARCH COMBINED WITH MEDICAL CARE

31. The physician may combine medical research with medical care only to the extent that the research is justified by its potential preventive, diagnostic or therapeutic value and if the physician has good reason to believe that participation in the research study will not adversely affect the health of the patients who serve as research subjects.
32. The benefits, risks, burdens and effectiveness of a new intervention must be tested against those of the best current proven intervention, except in the following circumstances:

    • The use of placebo, or no treatment, is acceptable in studies where no current proven intervention exists; or

    • Where for compelling and scientifically sound methodological reasons the use of placebo is necessary to determine the efficacy or safety of an intervention and the patients who receive placebo or no treatment will not be subject to any risk of serious or irreversible harm. Extreme care must be taken to avoid abuse of this option.
33. At the conclusion of the study, patients entered into the study are entitled to be informed about the outcome of the study and to share any benefits that result from it, for example, access to interventions identified as beneficial in the study or to other appropriate care or benefits.
34. The physician must fully inform the patient which aspects of the care are related to the research. The refusal of a patient to participate in a study or the patient’s decision to withdraw from the study must never interfere with the patient-physician relationship.
35. In the treatment of a patient where proven interventions do not exist or have been ineffective, the physician, after seeking expert advice, with informed consent from the patient or a legally authorized representative, may use an unproven intervention if in the physician's judgment it offers hope of saving life, re-establishing health or alleviating suffering. Where possible, this intervention should be made the object of research, designed to evaluate its safety and efficacy. In all cases, new information should be recorded and, where appropriate, made publicly available.
